# Direct Imaging of Chirality Transfer Induced by Glycosidic Bond Stereochemistry in Carbohydrate Self-Assemblies

**DOI:** 10.1021/jacs.4c16088

**Published:** 2025-03-06

**Authors:** Shuning Cai, Joakim S. Jestilä, Peter Liljeroth, Adam S. Foster

**Affiliations:** † Department of Applied Physics, Aalto University, Espoo 00076, Finland; ‡ WPI Nano Life Science Institute (WPI-NanoLSI), Kanazawa University, Kakuma-machi, Kanazawa 920-1192, Japan

## Abstract

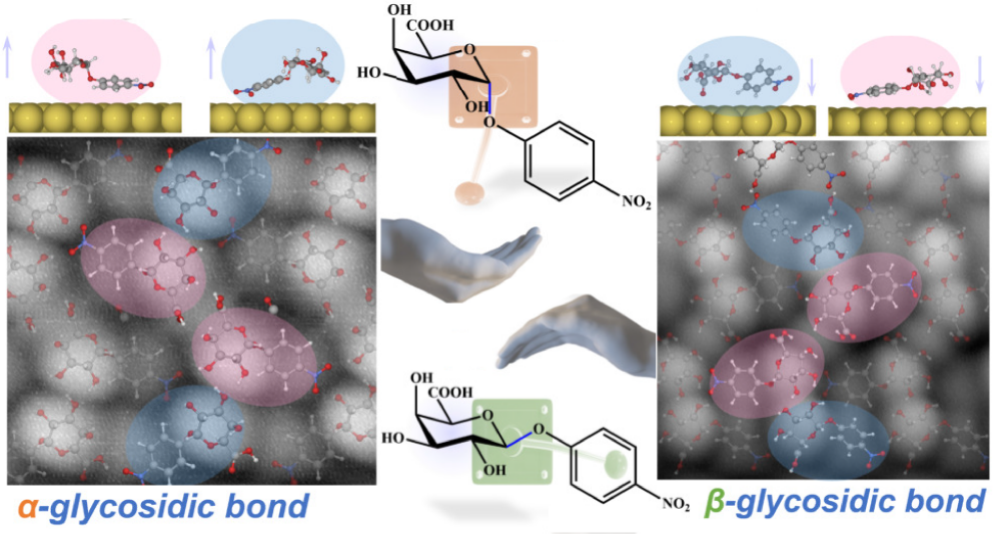

Carbohydrates, essential biological building blocks, exhibit functional mechanisms tied to their intricate stereochemistry. Subtle stereochemical differences, such as those between the anomers maltose and cellobiose, lead to distinct properties due to their differing glycosidic bonds; the former is digestible by humans, while the latter is not. This underscores the importance of precise structural determination of individual carbohydrate molecules for deeper functional insights. However, their structural complexity and conformational flexibility, combined with the high spatial resolution needed, have hindered direct imaging of carbohydrate stereochemistry. Here, we employ noncontact atomic force microscopy integrated with a data-efficient, multifidelity structure search approach accelerated by machine learning integration to determine the precise 3D atomic coordinates of two carbohydrate anomers on Au(111). We observe that the stereochemistry of the glycosidic bond regulates on-surface chiral selection in carbohydrate self-assemblies. The reconstructed models, validated against experimental data, provide reliable atomic-scale structural evidence, uncovering the origin of the on-surface chirality from carbohydrate anomerism. Our study confirms that nc-AFM is a reliable technique for real-space discrimination of carbohydrate stereochemistry at the single-molecule level, providing a pathway for bottom-up investigations into the structure–property relationships of carbohydrates in biological research and materials science.

## Introduction

Carbohydrates, the most prevalent biomolecules, exhibit chirality, a fundamental property in chemistry, biology, physics, and material science, across multiple scales, ranging from sub- to supramolecular levels. Chirality conferral plays a critical role in the synthesis of chiral nanostructures with unusual optical and magnetic properties, as well as novel pharmaceutical compounds.^1,2^ However, the mechanism by which chirality transfers from individual molecules to self-assembled structures remains elusive.^3−5^ Despite the fundamental importance of glycosidic bond stereochemistry in dictating biological processes such as enzyme recognition and cellular communication, its role in propagating chirality during self-assembly processes remains unexplained and lacks definitive structural evidence.^6^ Carbohydrate function is highly dependent on subtle structural features, as the three-dimensional arrangement of atoms in carbohydrates is closely linked to their intrinsic stereoelectronic effects, including orbital interactions that govern both structure and function.^7,8^ This emphasizes the need for precise structural determination at the submolecular level for unraveling the functional mechanisms of carbohydrates.

The myriad of possible regio- and stereochemical combinations among more than 100 known types of monosaccharides, combined with their extensive conformational flexibility, result in numerous possible isomers and intricate assembly rules for carbohydrate-based materials. The structural diversity not only offers carbohydrates high information density and a broad spectrum of tunable functionalities in both biological processes and synthetic materials^9,10^ but also complicates stereochemical control in carbohydrate synthesis and introduces substantial obstacles for structural characterization. This has contributed to the slower progress of the glycomics field compared to the rapid advancements in genomics and proteomics.^11,12^ Established analytical techniques such as X-ray crystallography (XRD), cryogenic transmission electron microscopy (cryo-TEM), and nuclear magnetic resonance (NMR) face significant constraints when applied to carbohydrates due to poor crystallization, susceptibility to radiation damage (in TEM), and broad overlapping spectral signals resulting from the coexistence of multiple conformations.^13−15^ Recent advances in scanning tunneling microscopy (STM) have achieved submolecular resolution real-space observation of individual carbohydrate molecules,^16−18^ opening new avenues for structural analysis of carbohydrates at the single-molecule level. Nevertheless, subnanometer resolution remains insufficient for fully resolving carbohydrate stereostructures. Providing the highest spatial resolution among real-space techniques, nc-AFM with a functionalized tip holds untapped potential for revealing stereochemical and conformational differences of individual carbohydrates.^19−21^ Functionalizing the metal tip with a single, relatively inert atom or molecule (such as CO, Xe, or pentacene) enables very short tip–sample distances. As the tip approaches the surface, Pauli repulsion increasingly dominates, resulting in sharper image features. This allows for more precise sampling of molecular edges and for resolving individual chemical bonds, offering detailed insights into their structural configurations.^22^

As a surface analysis technique, nc-AFM is primarily applied to planar molecules and requires efficient structure search or reconstruction approaches when imaging nonplanar molecules like carbohydrates, due to the nonintuitive images typically obtained when imaging 3D systems. While attempts have been made to employ machine learning approaches to infer 3D molecular structures directly from AFM images,^22,23^ these as yet remain too limited in accuracy when faced with the complexity of carbohydrate systems. To this end, we employ a data-efficient multifidelity global optimization protocol that merges active learning with density functional theory (DFT), which simultaneously provides training data for a machine learning interatomic potential (MLIP), which is then used to expand the number of candidate structures by accelerating their evaluation. As a result, here, we demonstrate the real-space observation of carbohydrate stereochemistry in supramolecular assemblies with atomic-scale resolution by leveraging the aforementioned structure search protocol validated by matching simulated and experimental constant-current STM and height-dependent nc-AFM images. The precise atomic positions of our validated models enable correlating the distinct on-surface chiralities of the self-assemblies with the stereoelectronic properties of the individual molecular building blocks, providing a rare example of homochiral self-assemblies induced by carbohydrate anomerism. Our work provides a bottom-up approach to studying structure–property relationships in carbohydrate molecules, opening an avenue for the development of carbohydrate-based supramolecules, ultimately shedding light on the design of complex molecular architectures.

## Results and Discussion

### The Role of Anomerism in Carbohydrate Self-Assembly on Au(111) Surfaces

**Figure 1:**
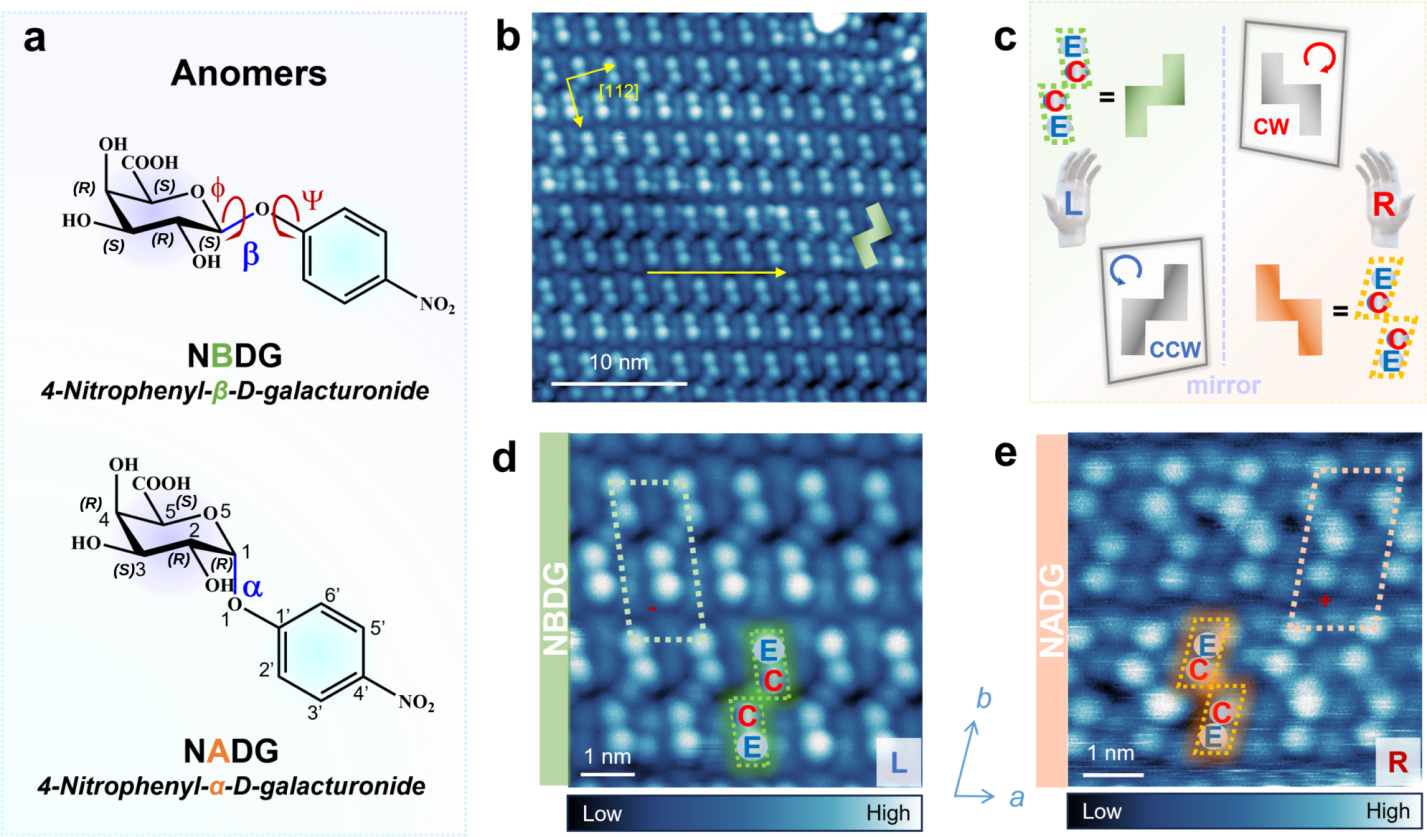
Influence of anomerism on chiral selection in carbohydrate self-assembly on Au(111). (**a**) Chemical structure of 4-nitrophenyl-α-d-galacturonide (**NBDG**, top) and 4-nitrophenyl-β-d-galacturonide (**NADG**, bottom), which differ in the orientation of the glycosidic bond (C_1_–O_1_) highlighted in navy, marked with α and β symbols, respectively. The *R*/*S*-configurations of the chiral centers are marked to emphasize the opposite handedness of the anomeric carbons (C_1_) of the two anomers. (**b**) High-resolution STM topography probed by a CO-functionalized tip of the **NBDG** self-assembly. Set point: 200 mV and 5 pA. (**c**) Schematic representation of the chiral relationship of the **NBDG** and the **NADG** lattice basic unit. Zoomed-in high-resolution STM topography of **NBDG** (**d**) and **NADG** (**e**) self-assemblies. Set point: 200 mV, 5 pA; 247 mV, 5.76 pA, respectively.

To investigate the impact of carbohydrate stereochemistry on 2D crystallization, we studied two carbohydrate molecules: 4-nitrophenyl-β-d-galacturonide (**NBDG**) and 4-nitrophenyl-α-d-galacturonide (**NADG**), as depicted in the chemical structures in Figure 1a. The two molecules were deposited sequentially on the Au(111) surface. Both **NBDG** and **NADG** possess the same monosaccharide backbone and nitrophenyl substituent marked in lilac and cyan, respectively. The key difference between the two molecules is the connectivity and orientation of the glycosidic bond (C_1_–O_1_, highlighted in navy), which is part of the linkage connecting the two subunits, resulting in their classification as anomers. This means that while the investigated molecules do possess enantiomers (these are the corresponding l-galacturonide anomers), they are not an enantiomeric pair. In the β-anomer, the exocyclic oxygen (O_1_) is bonded to the anomeric carbon (C_1_) in the equatorial position, whereas in the α-anomer, the attachment is in the axial position. Both anomers have five chiral centers, of which the configuration differs only for C_1_. In the α-anomer, the configuration is right-handed (*R*), while in the β-anomer, it is left-handed (*S*). The remaining chiral centers are identical and designated (*2R*, *3S*, *4R*, and *5S*). It is important to note that the C_1_–O_1_ and O_1_–C_1_′ bonds, along with the −OH and −COOH groups in the monosaccharide unit, are rotatable, which enables multiple conformations.

Following the electrospray deposition (ESD) of **NBDG** from ambient conditions into UHV at room temperature, ordered homochiral superstructures emerge on the Au(111) surface (Figures 1b and S1). We notice that the growth orientation of the **NBDG** self-assemblies shows a weak correlation with the lattice directions of the Au(111) surface. This is suggested by the angles between the *a*-axis of the unit cell and the [112̅] direction of the substrate across two different domains, as illustrated by the yellow arrow, which are −15.5 ± 1° and +35.5 ± 1° (see Figure S1). In the high-resolution STM probed by a CO tip (Figure 1b), we observe that the basic unit of the lattice consists of four bright protrusions, depicted in a green Z-shape pattern. In the enlarged high-resolution STM image (Figure 1d), the two central protrusions, labeled **C**, are identical to each other but differ from the edge protrusions, labeled **E**, the latter two also being identical to each other.

In a similar fashion, **NADG** forms regular self-assemblies on Au(111) upon deposition at room temperature, as shown in Figures 1e and S2. In contrast, the growth orientation of the **NADG** self-assemblies appears to correlate with the lattice directions. Different domains exhibit the *a*-axis vector aligning either along the [112̅] direction or forming a 30° angle with it (see Figure S2 for more details). The basic unit of the **NADG** lattice, depicted within the orange frame, also comprises two pairs of bright protrusions. Like the **NBDG** basic unit, the central and edge protrusions, denoted as **C** and **E** respectively, are mutually identical. Unlike the negative angle observed in the **NBDG** unit cell, the angle between the *a*-axis relative to the *b*-axis is positive in the **NADG** lattice (see Figure S2 for further details). The Z(S)-shaped basic **NADG**/**NBDG** unit exhibits *C*_2_ point group symmetry, while nonsuperimposability with the mirror image indicates chirality, as illustrated in Figure 1c. Moreover, the basic unit of **NADG** exhibits right-handedness (*R*), whereas that of **NBDG** displays left-handedness (*L*), matching the local chiral environment around the anomeric carbon atoms. It should be noted that although the **NADG**/**NBDG** assemblies appear as mirror images owing to the low STM resolution, they cannot be surface enantiomers due to the diastereomeric relation of the constituent molecules. The propagation of the chiral **NADG**/**NBDG** basic unit throughout the lattice imparts consistent chirality to the entire superstructure. The observation of only one type of on-surface chirality for a given anomer demonstrates preference toward one type of handedness. Chiral selection is a process in which one handedness is preferentially expressed over its mirror image. In the absence of such selection, one might expect to see a mixture of assemblies with differing handedness following deposition, as observed when prochiral succinic acid molecules adsorb onto Cu(110),^24^ or even achiral structures, as exemplified by one of the organizational phases of the chiral molecule alanine.^25^ However, since we did not observe self-assemblies of the opposite chirality when depositing each individual anomer, our results demonstrate spontaneous chiral selection on an achiral surface. To clarify, when referring to the opposite chirality of the assemblies, we refer to structures that were not observed in our experiments. Finally, our observations provide an example of hierarchical chirality, arising from the coexistence of multiple kinds of molecular chirality in a system, where the zero-dimensional chirality is transferred from the anomeric carbon—the one chiral center having opposite configurations in the deposited molecules—to the two-dimensional on-surface chirality of their assembled structures.^26^

### Reconstruction of 3D Atomic-Resolution Structural Models

**Figure 2:**
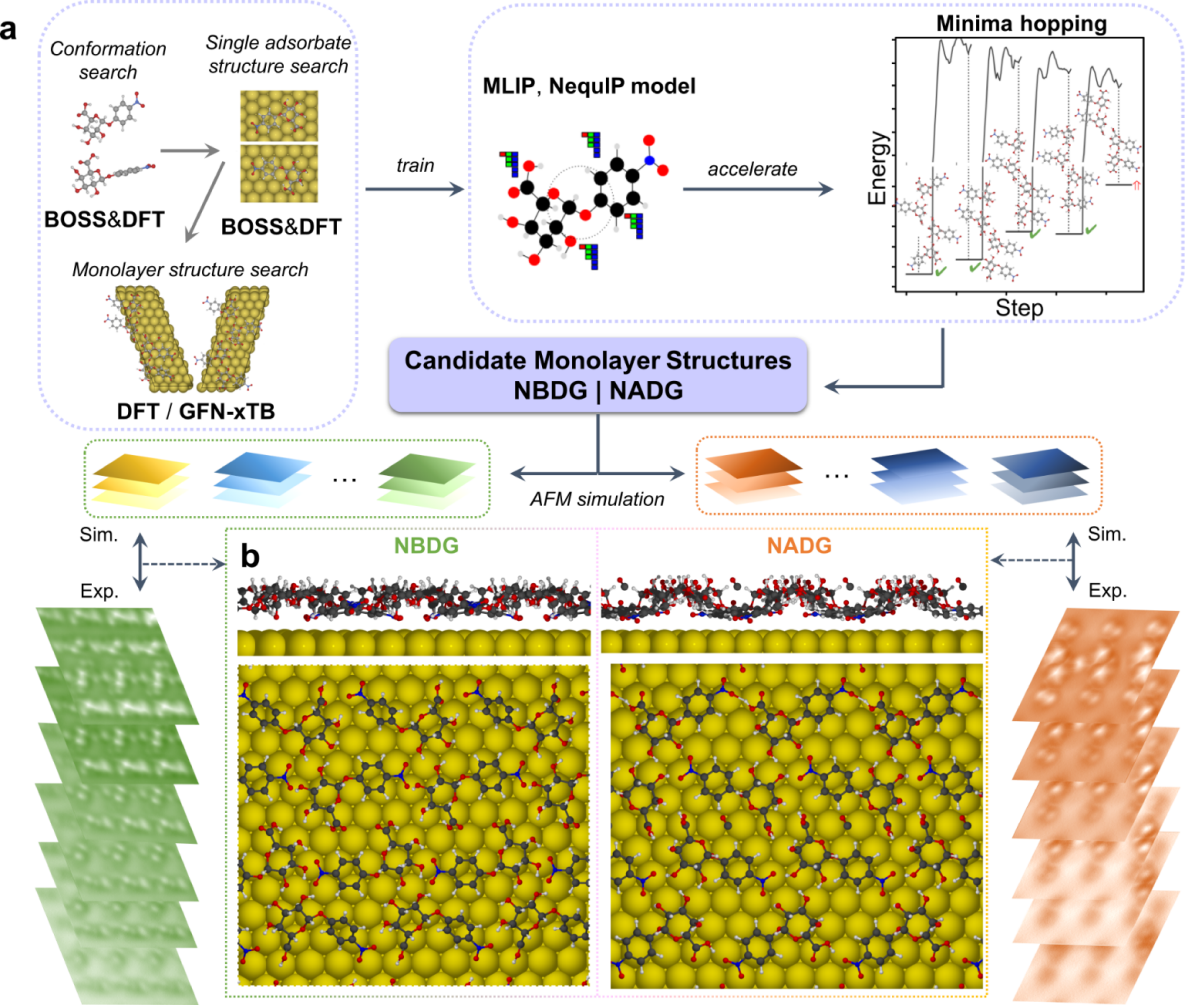
Workflow of 3D atomic-resolution structural model reconstruction. (**a**) Monolayer adsorption configuration structure search employing a multifidelity modeling approach (left to right): the search begins by identifying the most stable conformers and adsorption configuration for single-molecule **NBDG**/**NADG** adsorbates, which is achieved using a surrogate model of the DFT potential energy surface constructed via BOSS to accelerate the identification of the global minima. Initial monolayer structures are manually constructed and relaxed using high-fidelity DFT, supplemented by high-temperature molecular dynamics snapshots from the GFN-xTB method. The aforementioned acquired data are subsequently used to train a MLIP, NequIP. The trained potential accelerates the exploration of possible structures with minima hopping, providing a set of candidate monolayer structures. (**b**) The final monolayer structures of **NBDG** and **NADG** are obtained by manually adjusting the global minimum structures from the minima hopping procedure until the simulated AFM images resemble the experimental counterparts, the resulting structures being the most consistent with the experimental results.

To resolve the underlying atomic-resolution structures of the **NBDG** and **NADG** self-assemblies, we conducted height-dependent AFM measurements with a CO-functionalized tip (Figure 2). During the initial structural screening, we observed that even small changes in the 3D monolayer structures of **NBDG**/**NADG** are distinctly reflected in the simulated AFM images, demonstrating the potential of AFM to guide detailed structural investigations.

Achieving agreement between a structural model and experimental data requires extensive exploration of possible structures, in particular, when considering a full monolayer consisting of flexible 3D molecules. To address the labor-intensive construction of monolayer structures and the computational cost of subsequent DFT computations, we employ the minima hopping algorithm,^27^ integrated with a state-of-the-art MLIP, the highly data-efficient Neural Equivariant Interatomic Potential (NequIP).^28^ A simplified schematic of the workflow of the 3D atomic-resolution structural model reconstruction is presented in Figure 2. Both the conformational search and the adsorption configuration search for an individual **NBDG**/**NADG** molecule are performed on a surrogate model of the DFT potential energy surface (PES), constructed with the Bayesian Optimization Structure Search (BOSS) package.^29^ Initial monolayer structures are manually built using the most stable single-molecule adsorption configurations, guided by the lattice parameters derived from experimental STM images (Figures S3, S4 and S9), and then relaxed using DFT. The aforementioned data are supplemented with high-temperature molecular dynamics snapshots from the semiempirical tight-binding DFT method GFN-xTB,^30^ which are then used to train the NequIP model. While BOSS is limited to less than 20 degrees of freedom, making it insufficient for full monolayer structure establishment, minima hopping using the trained NequIP model overcomes this limitation, accelerating the sampling process without compromising computational accuracy. This multifidelity modeling approach was used to obtain 850 candidate monolayer structures for the two anomers. The simulated AFM stacks of the global minimum structures are, overall, close to the experimental results and show better agreement than candidates corresponding to higher energies. The final reconstructed 3D monolayer structures of **NBDG** and **NADG** (Figure 2b) are obtained by refining the atomic positions of the global minimum structures, followed by final constrained DFT relaxations.

**Figure 3:**
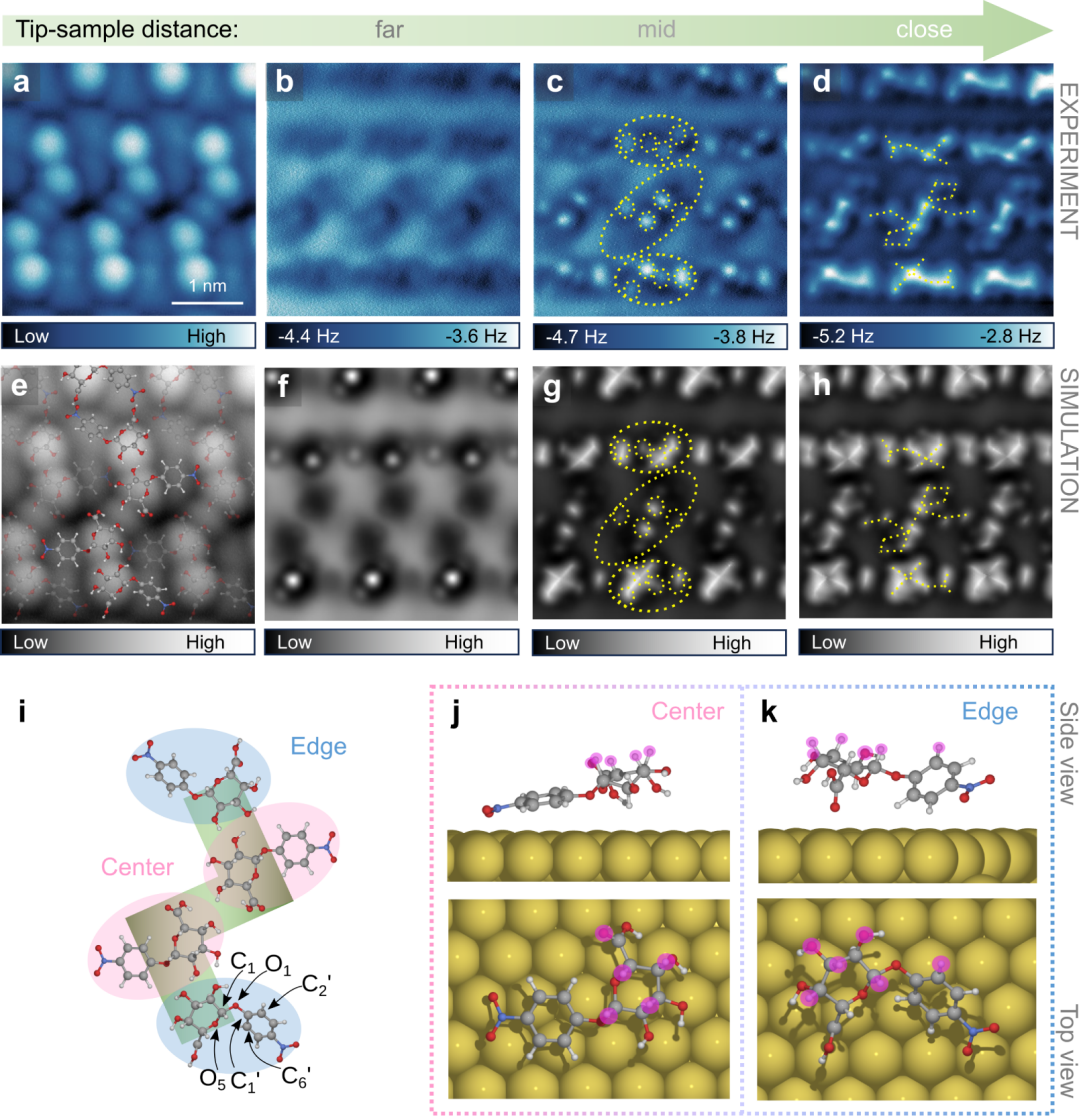
STM and height-dependent AFM images of the **NBDG** self-assembly. (**a**, **e**) High-resolution CO-tip STM images covering the same area as in the height-dependent AFM stack (**a**), the second image showing the superimposed 3D reconstructed molecular structure (**e**). Set point: 200 mV, 5 pA. (**b**–**d**) Constant-height AFM images at different tip–sample distances, far: −80 pm (**b**); mid: −50 pm (**c**); and close: −20 pm (**d**). The tip height values are relative to the STM set point (200 mV, 5 pA). (**f**–**h**) The simulated AFM images approximately corresponding to the experimental far (**f**), mid (**g**), and close (**h**) tip–sample distances. (**i**–**k**) The absolute configuration of the **NBDG** basic unit, displaying two distinct conformations: the central (pink) and the edge conformations (blue) (**i**); the side view (upper panels) and top view (lower panels) of the individual central (**j**) and edge (**k**) conformations adsorbed on Au(111). Yellow dashed lines are added to guide eye toward matching features in the experimental and simulated images. The atoms contributing directly to the image contrast are marked by purple circles.

**Figure 4:**
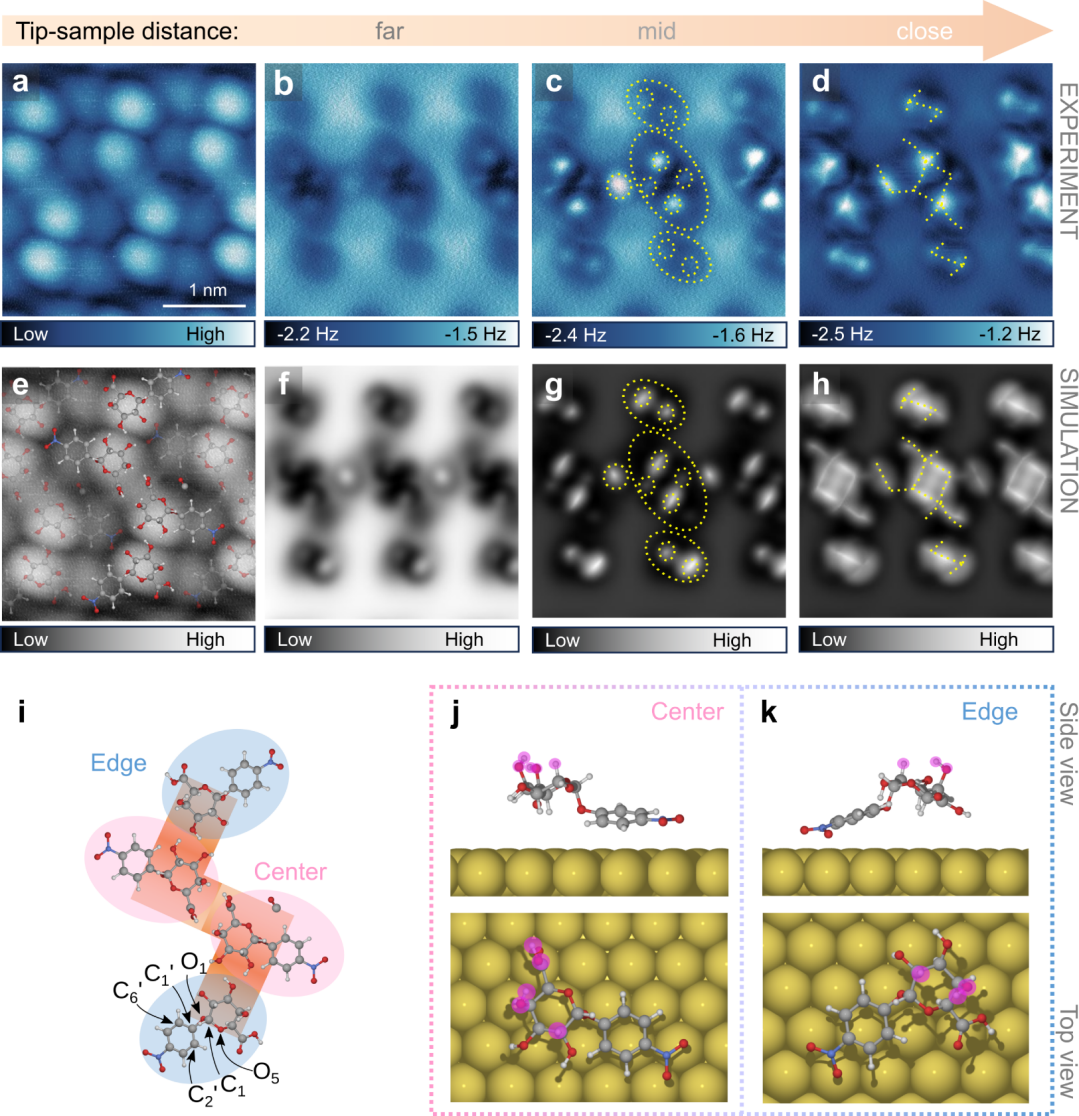
STM and height-dependent AFM images of the **NADG** self-assembly. (**a**, **e**) High-resolution CO-tip STM images covering the same area as in the height-dependent AFM stack (**a**), the second image showing the superimposed 3D reconstructed molecular structure (**e**). Set point: 247 mV, 5.76 pA. (**b**–**d**) Constant-height AFM images at different tip–sample distances, far: −90 pm (**b**); mid: −70 pm (**c**); and close: −50 pm (**d**). The tip height values are relative to the STM set point (247 mV, 5.76 pA). (**f**–**h**) The simulated AFM images approximately corresponding to the experimental far (**f**), mid (**g**) and close (**h**) tip–sample distances. (**i**–**k**) The absolute configuration of the **NADG** basic unit, displaying two distinct conformations: the central (pink) and the edge conformations (blue) (**i**); the side view (upper panels) and top view (lower panels) of the individual central (**j**) and edge (**k**) conformations adsorbed on Au(111). Yellow dashed lines are added to guide eye toward matching features in the experimental and simulated images. The atoms contributing directly to the image contrast are marked by purple circles.

The simulated AFM stacks at different tip–sample distances based on the reconstructed 3D models of the **NBDG** and **NADG** molecules (Figures 3f–h and 4f–h) closely align with the corresponding experimental results, supporting the validity of the model structures. Overlaying the **NBDG** and **NADG** structures onto their STM images (Figures 3a,e and 4a,e), captured in the same area as the AFM stacks, reveals that both the left-handed **NBDG** basic unit and the right-handed **NADG** basic unit comprise four molecules with two distinct conformations: denoted as the central and edge conformations, which are highlighted in pink and blue, respectively (Figures 3i and 4i). The bright protrusions observed in the STM images are primarily attributed to the pyranose rings of the **NBDG** and **NADG** molecules. In both the central and edge conformations for the **NBDG** basic unit, the −O_3_H, −O_4_H, and −COOH groups in the pyranose ring are oriented toward the Au(111) surface, as clearly seen in the individual adsorption configurations from the top and side views (Figure 3j,k). In contrast, for the **NADG** basic unit, these same groups are oriented away from the Au(111) surface, while the glycosidic bond (C_1_–O_1_) remains oriented toward the surface, similar to the **NBDG** conformations, leading to a more parallel alignment between the nitrophenyl group and the surface (Figure 4j,k). Additionally, DFT calculations indicate that the rotational barriers of the −OH groups in the central units of the **NADG** assembly are relatively low, around 10 kJ/mol at their minimum (Figure S17), compared to the more rigid hydrogen atoms in the pyranose ring. This agrees with our experimental observations, suggesting that the −OH groups are more susceptible to movement due to tip–sample interactions. At closer tip–sample distances, the simulated AFM images for **NBDG** show better agreement with experimental data, as the contrast features predominantly arise from the rigid hydrogen atoms (Figure 3j,k, side view). In contrast, for **NADG**, the −OH groups significantly contribute to the contrast features (Figure 4j,k, side view), leading to deviations between the simulated and experimental images at close tip–sample distances. These deviations arise from changes in the atomic positions of the more flexible −OH groups caused by the tip–sample interactions, which are not accounted for in the AFM simulations (more details in Figure S17).

Notably, despite the presence of multiple on-surface configurations, the molecules exhibit the same on-surface chirality in the self-assemblies of both **NBDG** and **NADG**, as indicated by the opposite orientation of the −O_3_H, −O_4_H, and −COOH groups in the pyranose rings relative to the surface, as well as the similar orientation of the exocyclic oxygen (O_1_) toward the substrate. These groups tend to follow a similar hydrogen-bonding pattern, resulting in the molecules being arranged in an alternating orientation sequence within the basic unit on the surface. This reveals that both **NBDG** and **NADG** molecules in their respective hydrogen-bonded self-assemblies exhibit chiral-selective adsorption, leading to left-handedness and right-handedness in the basic units and overall superstructures, respectively.

### Stereoelectronic Effects of Individual Carbohydrate Molecules on the Assembled Structures

**Figure 5:**
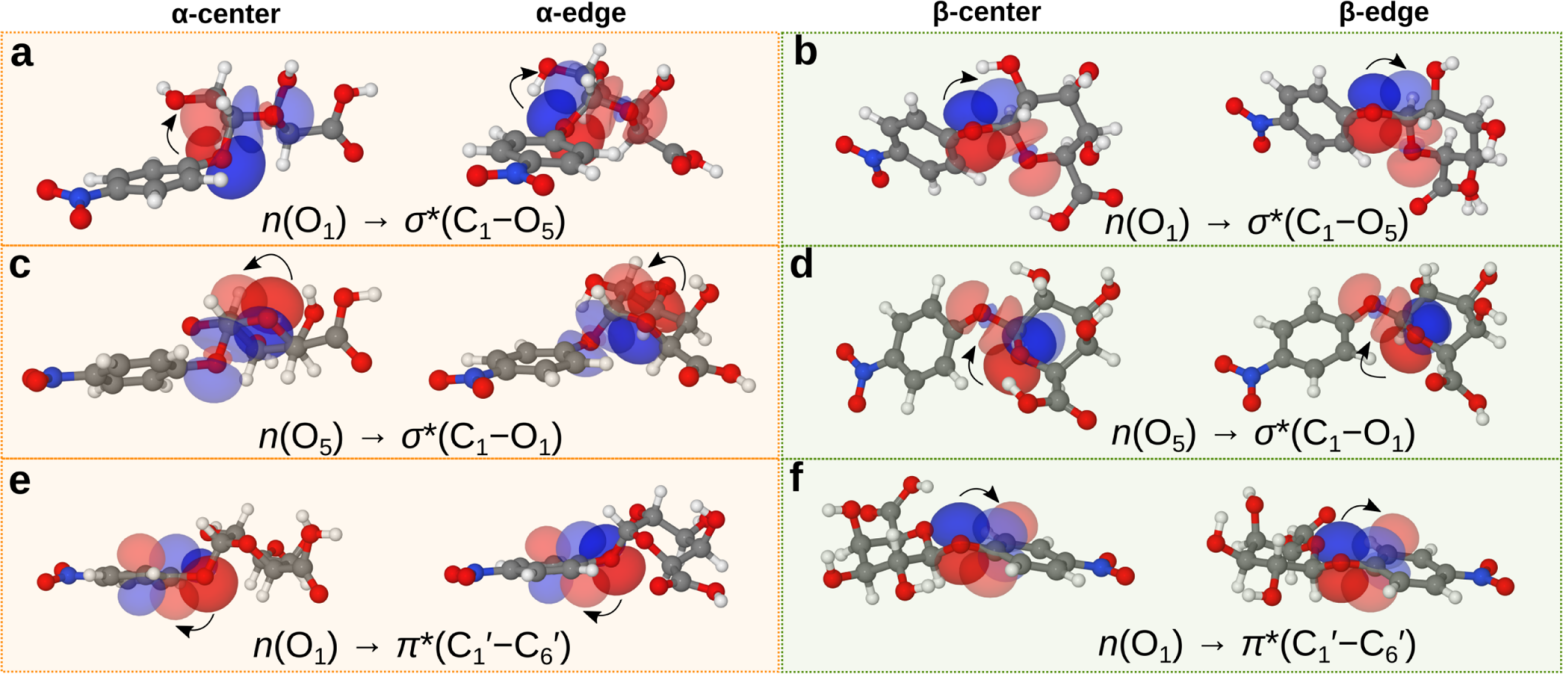
Selected donor–acceptor orbitals relevant to the stereoelectronics of individual molecules in the **NADG**/**NBDG** assemblies. Donor–acceptor orbital pairs are superimposed onto the same molecule, with the empty acceptor orbitals being slightly more translucent than the filled donor orbitals, and the electron flow direction is indicated with curved arrows. **NADG** structures are displayed with orange background, **NBDG** with green background, both showing the central (left) and edge conformations (right), respectively. (**a**, **b**) Donor–acceptor interactions related to the exo-anomeric effect for **NADG** (**a**) and **NBDG** (**b**). (**c**, **d**) Donor–acceptor interactions related to the endo-anomeric effect for **NADG** (**c**) and **NBDG** (**d**). (**e**, **f**) Donor–acceptor interactions related to the donation of electron density to the nitrophenyl substituent for **NADG** (**e**) and **NBDG** (**f**).

Having obtained precise structural models of the monolayers, we are in a position to discern their intrinsic stereoelectronic effects. This is achieved through natural bond orbital (NBO) analysis, shedding light on the intra- and intermolecular interactions underlying the observed chiral selection. The analysis (Figure 5 and Table 1) reveals vital aspects of orbital interactions related to stereoelectronic effects, such as those related to the endo- and exo-anomeric effects, which play an important role in determining the molecular structure of carbohydrates. These two effects correspond to *n*(O_5_) → σ*(C_1_–O_1_) and *n*(O_1_) → σ*(C_1_–O_5_) hyperconjugative interactions, respectively.^31^ Generally, a donor–acceptor interaction with a higher energy has a larger effect on the overall molecular structure than interactions with lower energies. In **NADG**, the endo-anomeric effect dominates in both the α-center and α-edge conformations (Figure 5c). However, the relative contribution of the exo-anomeric effect increases substantially in the α-edge, as indicated by the reduced energy difference between the two hyperconjugative interactions (Δ*E* = 35.7 and 16.3 kJ/mol, for α-center and α-edge, respectively). A dihedral angle change of 44.5° (O_5_–C_1_–O_1_–C_1_′) in going from α-center to α-edge further supports improved orbital alignment between *n*(O_1_) and σ*(C_1_–O_5_) (Figure 5a). Additionally, in the α-center, the C_1_–O_5_ bond is 0.06 Å shorter than C_1_–O_1_, whereas this same bond length difference reduces to 0.02 Å in the α-edge, in line with the more balanced contributions of endo- and exo-anomeric effects.

**Table 1:** Selected Computational Bond Distances in Å, Dihedral Angles ϕ and ψ in Degrees, and Donor–Acceptor Interaction Energies from Second-Order Perturbation Theory in the NBO Basis for the **NADG**/**NBDG** Adsorbates in kJ/Mol

	α-center	α-edge	β-center^a^	β-edge^a^
*d*(C_1_–O_5_)	1.41	1.42	1.44	1.42
*d*(C_1_–O_1_)	1.47	1.44	1.41	1.42
ϕ (∠O_5_–C_1_–O_1_–C_1_′)	134.5	90.0	279.1	294.5
ψ (∠C_1_–O_1_–C_1_′–C_6_′)	93.7	125.4	225.1	217.8
**exo:***n*_(O1)_ → σ*_(C1–O5)_	24.1	49.2	57.9	47.3
**endo:***n*_(O5)_ → σ*_(C1–O1)_	59.8	65.6	15.4	16.4
Σρ(*r*) → σ*_(C1–O5)_	58.9	82.0	93.6	81.0
Σρ(*r*) → σ*_(C1–O1)_	118.7	111.9	67.5	75.3
*n*_(O1)_ → σ*_(C1′–C2′)_	27.9	14.8	10.0	10.1
*n*_(O1)_ → σ*_(C1′–C6′)_	28.9	31.9	32.3	33.8
*n*_(O1)_ → π*_(C1′–C6′)_	32.8	56.2	74.3	87.4

a **NBDG** parameters from fully relaxed structure.

In **NBDG**, the exo-anomeric effect dominates for both the β-center and β-edge conformations, while the endo-anomeric effect is deactivated in the equatorial conformation due to the misalignment between the *n*(O_5_) lone pair and the σ*(C_1_–O_1_) orbital, as indicated by the ϕ dihedral angles (Figure 5b,d). The interaction energies reflect this deactivation, with the exo-anomeric effect providing the most favorable interactions at 57.9 and 47.3 kJ/mol in the β-center and β-edge, respectively, compared to the endo-effect at 15.4 kJ/mol (Δ*E* = 42.5 kJ/mol) and 16.4 kJ/mol (Δ*E* = 30.9 kJ/mol). Notably, the C_1_–O_5_ and C_1_–O_1_ bond length difference is less pronounced in **NBDG** compared to **NADG**, particularly for the β-edge conformation, where both bonds measure 1.42 Å. The endo- and exo-anomeric effects alone cannot explain this, as the difference in interaction energies here is comparable to that of the α-edge, which *does* display different C_1_–O_5_ and C_1_–O_1_ bond lengths. This discrepancy can be understood by including the sum of interaction energies between all donors and the corresponding antibonding orbitals in the analysis. Since these bond distances are equal, we should expect a minor difference in the total interaction energies for the two antibonding orbitals, Σρ(*r*) → σ*(C_1_–O_5_) and Σρ(*r*) → σ*(C_1_–O_1_). The total donation to σ*(C_1_–O_5_) is larger in the β-center (Δ*E* = 12.6 kJ/mol), while the donation to σ*(C_1_–O_1_) is smaller (Δ*E* = −7.8 kJ/mol) than that in the β-edge, increasing the interaction energy difference between the two antibonding orbitals (Δ*E* = 26.1 kJ/mol) compared to the β-edge (Δ*E* = 5.7 kJ/mol). In contrast, the interaction energy difference is even more pronounced in **NADG**, particularly in the α-center, reaching up to 59.8 kJ/mol, aligning with the greater variation in C_1_–O_5_ and C_1_–O_1_ bond lengths. Furthermore, the aromatic nitrophenyl ring, which can interact with the lone pair orbitals on the glycosidic oxygen O_1_ through the π* and σ* orbitals, can influence the total interaction energies and the charge distribution.^32^ The total donation from *n*(O_1_) to the phenyl ring is more pronounced in **NBDG**, with the primary contribution arising from the interaction with the π(C_1_′–C_6_′) orbital, especially in the β-edge conformation from *n*(O_1_) → π*(C_1_′–C_6_′) at 87.4 kJ/mol (Figure 5f). The same interaction is also significant in the β-center at 74.3 kJ/mol, compared to the lower values in the α-center and α-edge, at 32.8 and 56.2 kJ/mol, respectively (Figure 5e). This pattern aligns with the Σρ(*r*) → σ(C_1_–O_5_) and Σρ(*r*) → σ*(C_1_–O_1_) interaction energies and corresponding bond length variations. The interactions between the *n*(O_1_) lone pair with the nitrophenyl group compete with those between the *n*(O_1_) lone pair and the C_1_–O_5_, counteracting the latter by providing an additional pathway for electron density transfer. This results in a more balanced charge distribution and a reduced difference in the bond lengths. More details on the individual donor–acceptor orbitals are given in Figure S19.

According to the stereoelectronic effects, the more imbalanced charge distribution in the central conformations of both **NBDG** and **NADG** basic units suggests that they serve as stronger hydrogen bond donors and acceptors, forming intermolecular bonds stronger than those in the corresponding edge conformations. This rationalizes the presence of multiple conformations within the basic unit, as it enhances electron density delocalization, stabilizing the hydrogen-bonded structure. Furthermore, the enhanced electron density transfer from the *n*(O_1_) orbital into the aromatic nitrophenyl group in **NBDG** suggests increased rigidity of the molecule as seen through a more constant ψ dihedral angle and a weaker interaction with the Au(111) surface compared to **NADG**. This is supported by a longer average distance between the nitrophenyl groups and the surface for the former at 3.84 Å, compared to 3.44 Å for the latter. The molecules are physisorbed onto the surface, where the additional negative charge donated to the nitrophenyl group reduces the van der Waals interactions mainly responsible for the adsorption.^33,34^ As shown in the side views (Figures 3j and 4j), the nitrophenyl ring in the α-center lies mostly parallel to the surface, while in the β-edge, it is more tilted, indicating a stronger surface interaction in the α-center, consistent with the electron density transfer trend. This is corroborated by the computed charge density differences between the adsorbed system and the isolated monolayer and substrate, where the nitrophenyl group clearly received more charge density in **NBDG** than in **NADG** (Figure S20). Besides, the nitrophenyl ring in both central and edge conformations in **NADG** preferentially adsorbs atop Au atoms, whereas **NBDG** shows no specific adsorption site preference.

## Conclusions

Through our study, we have achieved the real-space observation of carbohydrate stereochemistry in supramolecular assemblies with atomic resolution and constructed consistent 3D models of these through a modeling protocol merging machine learning with well-established first-principles methods. Owing to these detailed structural models, we can link the structural and stereochemical properties of the individual carbohydrates to the on-surface hierarchical chiralities in their self-assemblies. NBO analysis and charge density computations reveal that the stereochemistry of the glycosidic bond directly influences charge transfer to the aromatic nitrophenyl groups. This charge transfer modulates interactions between the nitrophenyl groups and the surface, competing with those between the former and the monosaccharide backbone, ultimately affecting other intermolecular interactions that are part of controlling the self-assembly process, in particular, the hydrogen bonding. These competitive interactions control the adsorption selectivity of the individual carbohydrate molecules studied herein and contribute to chiral selection in the self-assembly. This finding provides new insights into chiral conferral in self-assembly and on-surface stereoselective catalysis of materials and compounds based on carbohydrates. Finally, we have elucidated the role of glycosidic bond stereochemistry in influencing the charge distribution of the groups attached to the nonanomeric carbon, an aspect less frequently studied compared to the well-understood anomeric effects.

## Methods

### Sample Preparation and ESD

The Au(111) single crystal, obtained from MaTeck, was cleaned through repeated Ne^+^ sputtering with a beam energy of 1000 eV and an ion current of 30 μA for 10 min, followed by annealing at approximately 450 °C for 5 min. Solutions of **NADG** and **NBDG** for ESD were prepared by dissolving the powders in a 1:1 (v/v) mixture of methanol and acetonitrile, resulting in a final concentration of ∼0.02 mmol/L. The flow rate during deposition was set to 955 μL/h. Positive ion mode was employed, and the voltage applied to the emitter ranged from ∼3000 to 3500 V over a duration of approximately 30 min. Throughout the deposition process, the sample was maintained at room temperature in a UHV chamber with a base pressure of 1 × 10^–9^ mbar.

### STM/AFM Measurements

All experiments were conducted on a combined noncontact AFM/STM system (Createc) equipped with a commercial qPlus sensor with a Pt/Ir tip (resonance frequency *f*_0_ ≈ 301 470 Hz and quality factor *Q* ≈ 77 648). The system operated with an oscillation amplitude of *A* = 50 pm at *T* ≈ 5 K in UHV conditions, with a base pressure of approximately 1 × 10^–10^ mbar.

### DFT

DFT computations were done in FHI-aims^35^ and Gaussian.^36^ The functional chosen for most of the computations was PBE augmented with the Tkatchenko–Scheffler dispersion correction parameterized for surfaces, termed PBE+vdW^surf^^37,38^ with light defaults and first-tier basis functions. We used a 1 × 1 × 1 Monkhorst–Pack grid to sample the Brillouin zone. No spin polarization was employed due to the closed-shell character of the carbohydrates. The surface slab was constructed using four layers of 12 × 4 Au in the fcc111 structure, where the two lowest layers were kept fixed. The unit cell dimensions were determined from the experimental line profiles, which were subsequently relaxed with DFT, resulting in monoclinic unit cells with dimensions (*a*, *b*, γ) = (11.71 Å, 29.96 Å, 111.1°) for the **NADG** monolayer and (*a*, *b*, γ) = (11.70, 30.01, 73.18°) for **NBDG**. With these lattice parameters, the underlying surface deviates from that of pristine Au(111), which could be attributed to variations in Au–Au distances resulting from herringbone reconstruction of the real surface. In particular, we note that the relaxed model surface shows varying Au–Au distances both in the *a* (4.97–5.00 Å) and *b* (2.92–2.94 Å) directions of the face-centered unit cell, which is consistent with their experimentally observed anisotropic shortening.^39^ To investigate donor–acceptor interactions, we used the Natural Bond Orbital analysis (NBO version 3.1)^40^ as implemented in Gaussian. Here, as this analysis was done on the monolayers without including the surface, we opted for the B3LYP functional^41,42^ combined with the correlation-consistent cc-pVDZ basis set for a more accurate description of the electronic structure of the molecules.

### BOSS

BOSS is a general-purpose Bayesian optimization code developed by the Computational Electronic Structure Theory (CEST) Group at the Aalto University and the University of Turku (gitlab.com/cest-group/boss). For our application, we installed the code in a virtual conda environment on the computing cluster and interfaced it with FHI-aims through a shell script, which calls for the program to compute the DFT energy of a structure. For individual molecules, the computed energy was then subtracted from that of an arbitrary conformation to provide a relative value, while for adsorption structures, adsorption energies were computed for the given adsorption structure with respect to the isolated substrate and an arbitrary conformation for the desorbed molecule. These energies were parsed to BOSS and used to construct the surrogate model PES. The conformers/adsorption structures were altered according to the selected degrees of freedom with a Python script called on prior to the DFT energy evaluation. A quasi-random Sobol sequence was used to initialize the data, and we made use of the GP-Lower Confidence Bound acquisition function with an increase in exploration (elcb). Standard periodic kernels were used for rotation and *xy*-translation, while radial basis functions (rbf) were used for the *z*-coordinate. Acquired data points were multiplied by leveraging surface symmetry and applying symmetry operations to the adsorbate at high-symmetry sites of the fcc111 surface. Convergence of the model was determined by checking whether the predicted global minimum stopped changing between iterations. Initially, we conducted a search for the global minimum conformers of the two **NADG** and **NBDG** anomers, the degrees of freedom including the full rotation of the hydroxyl groups, carboxylate group, the two bonds involved in the glycosidic linkage, as well as the ^1^C_4_ to ^4^C_1_ ring inversion (9D search), basing the PES surrogate model on 599 DFT data entries (DFT single-point energies and corresponding structures) for **NADG** and 405 for **NBDG**. Following the construction of the surrogate model, local minima were determined by relaxing the structures on the model PES within the given degrees of freedom, starting from each of the acquired data points on the model surface. Finally, all of the predicted BOSS conformers were relaxed using DFT, resulting in 83 and 107 unique conformers for **NADG** and **NBDG**, respectively. Following this, we used the global minimum conformer to search for the global adsorption minima. The surrogate model for the adsorption of single **NADG** molecules on the surface (6D search) was constructed out of 654 DFT data entries, while the **NBDG** counterpart was constructed out of 628 DFT data entries. Out of 244 unique **NADG** adsorption structures predicted by BOSS, we relaxed the lowest 10 using DFT. Similarly, for the **NBDG** structures, the number of unique structures predicted was 185, and of these, we relaxed 17 with DFT. We limited ourselves to a subset of the adsorption structures due to the high computational cost and time inherent to DFT, working under the assumption that the lowest energy predictions from BOSS would represent the lowest energy DFT-relaxed structures adequately.

### CREST

The Conformer-Rotamer Ensemble Sampling Tool (CREST, version 2.12)^43,44^ was employed as an auxiliary method to probe the accuracy of BOSS for the **NADG** conformer analysis. We were unable to use this to validate the adsorption structure search, as it is currently unavailable for periodic systems. The length of the metadynamics run was determined to be 98 ps to fully sample the conformational phase space based on a flexibility measure of 0.288. This resulted in 119 unique conformers, somewhat more than what was provided by BOSS, which is expected since CREST employs collective variables that allow for more flexibility in the ring configurations. Nonetheless, the two methods identify the same lowest energy conformer, providing support for the BOSS-based analysis.

### NequIP

We trained the MLIP Neural Equivariant Interatomic Potential (NequIP)^28^ on DFT data to accelerate the screening of candidate monolayer structures. The code (github.com/mir-group/nequip) was installed in a virtual environment with conda on the Aalto Science IT Triton cluster, where training was performed on a 32GB Tesla V100 GPU. NequIP is among the state-of-the-art within MLIPs, in particular, with respect to its high data efficiency resulting from its E(3)-equivariant design. Nonetheless, it should be mentioned that an improved version—BOTNet—has recently been described.^45^ The training data contained 2532 entries, including the atomic coordinates, along with their computed DFT energies and forces, randomly split into 2232 for training and 300 for validation. All energies were modified by subtracting the atomization energies from the total system energies. The data included a mixture of the conformers of the isolated molecules, single molecule adsorbates, a selection of monolayer structures for both **NADG** and **NBDG**, and finally structures sampled from high-temperature NVE molecular dynamics (*T* = 4000 K) using the semiempirical tight-binding DFT method GFN-xTB.^30^ Both the trained NequIP model and GFN-xTB were used as calculators in the Atomic Simulation Environment (ASE).^46^ We used NequIP version 0.5.6 along with e3nn^47^ version 0.4.4, PyTorch 1.13.0, and CUDA toolkit 11.7. The cutoff radius was 4.5 Å, training batch size 5, validation batch size 10, while the learning rate was set to 0.0075. The configuration file (*config.yaml*) containing all training parameters, the trained model itself, as well as the training dataset can be found in the Zenodo repository (DOI: 10.5281/zenodo.13990712).

### Minima Hopping

The minima hopping algorithm was used with the trained NequIP model, employing Hookean bond constraints to preserve intact molecules during the high-temperature NVE MD propagation steps of the algorithm. The velocity Verlet integrator was used to calculate the trajectories, and the initial velocities were set by a Maxwell–Boltzmann distribution. The energy criterion (*E*_diff_) for accepting a new minimum was initially set to 2.5 eV. The initial temperature (*T*_0_) was set to 4000 K in an attempt to sample the configurational phase space efficiently. However, the molecular adsorbates are easily desorbed at such high temperatures, so we added a Hookean volatilization constraint, i.e., a restorative force keeping the molecules from drifting further than 18 Å from the surface since this height allows full rotation of the molecules on the surface. This procedure has been previously described in the literature, and more details can be found in the original publication.^48^ All minimum structures from the procedure can be found in the Zenodo repository (DOI: 10.5281/zenodo.13990712).

### AFM Simulations

The final AFM images were simulated with the GPU version of the Probe-Particle Model^49,50^ using the Hartree potential of the sample and a *dz*^2^-multipole on the probe particle, mimicking a CO molecule at the apex of an SPM tip. The charge of the probe particle was set to −0.05, determined by the best apparent match with the experiment. Exploration and adjustment of structures were done by simulating AFM images using only the geometry as input, providing similar images containing the same main features as those with the more accurate simulations employing the Hartree potential.

### STM Simulations

STM images were simulated with FHI-aims, employing the Tersoff–Hamann approximation^51^ with a bias of 0.2 V. Visualization was done with Vesta and the WSxM software^52^ with isovalues typically kept in the range of 10^–10^ to 10^–12^ a.u. based on the match with experiment.

## Data Availability

Additional computational data including conformers, single adsorbate, and monolayer structures, along with trained NequIP potentials and data, can be found in the Zenodo repository (DOI: 10.5281/zenodo.13990712).

## Supporting Information Available

Experimental data in the form of large-scale STM images and topography line profiles. Moreover, it contains supplementary computational data from Bayesian Optimization Structure Search (BOSS), details from the minima hopping procedure, simulated large-scale AFM images and their overlap with experiment, comparison of experimental and simulated STM and AFM images for a subset of candidate structures, analysis of the rotational barriers for hydroxyl and carboxylate groups in the **NADG** assembly, as well as a summary of the orbital interactions relevant to the donor–acceptor orbital interactions in the assemblies (PDF)
