# Supplementary material for: Direct Imaging of Chirality Transfer Induced by Glycosidic Bond Stereochemistry in Carbohydrate Self-Assemblies
Source: J Am Chem Soc. 2025 Mar 6;147(11):9341–51. doi: 10.1021/jacs.4c16088 (PMC11926875; doi:10.1021/jacs.4c16088)
Supplement: Supplementary file 1 — ja4c16088_si_001.pdf [file ja4c16088_si_001.pdf]

Supporting Information: Direct imaging of chirality transfer induced by glycosidic bond stereochemistry in carbohydrate self-assemblies

Shuning Cai,[†,¶] Joakim S. Jestilä,[†,¶] Peter Liljeroth,[*,†] and Adam S. Foster[*,†,‡]

†*Department of Applied Physics, Aalto University, 00076 Aalto, Espoo, Finland*

‡*WPI Nano Life Science Institute (WPI-NanoLSI), Kanazawa University, Kakuma-machi, Kanazawa 920-1192, Japan*

¶*These authors contributed equally.*

E-mail: peter.liljeroth@aalto.fi; adam.foster@aalto.fi

Contents

Experimental **S-3**

Supplementary experimental data: large-scale STM images S-3

Supplementary experimental data: STM topography line profiles S-5

Computational **S-8**

Bayesian Optimization Structure Search (BOSS) S-8

Minima hopping for monolayer structures S-13

Large-scale AFM overlap S-18

Comparison of experimental and simulated STM and AFM images S-20

Rotational barriers of hydroxyl and carboxylate groups in the NADG assembly . . S-22
Orbital interactions S-26
Charge density differences S-27

Experimental

Supplementary experimental data: large-scale STM images

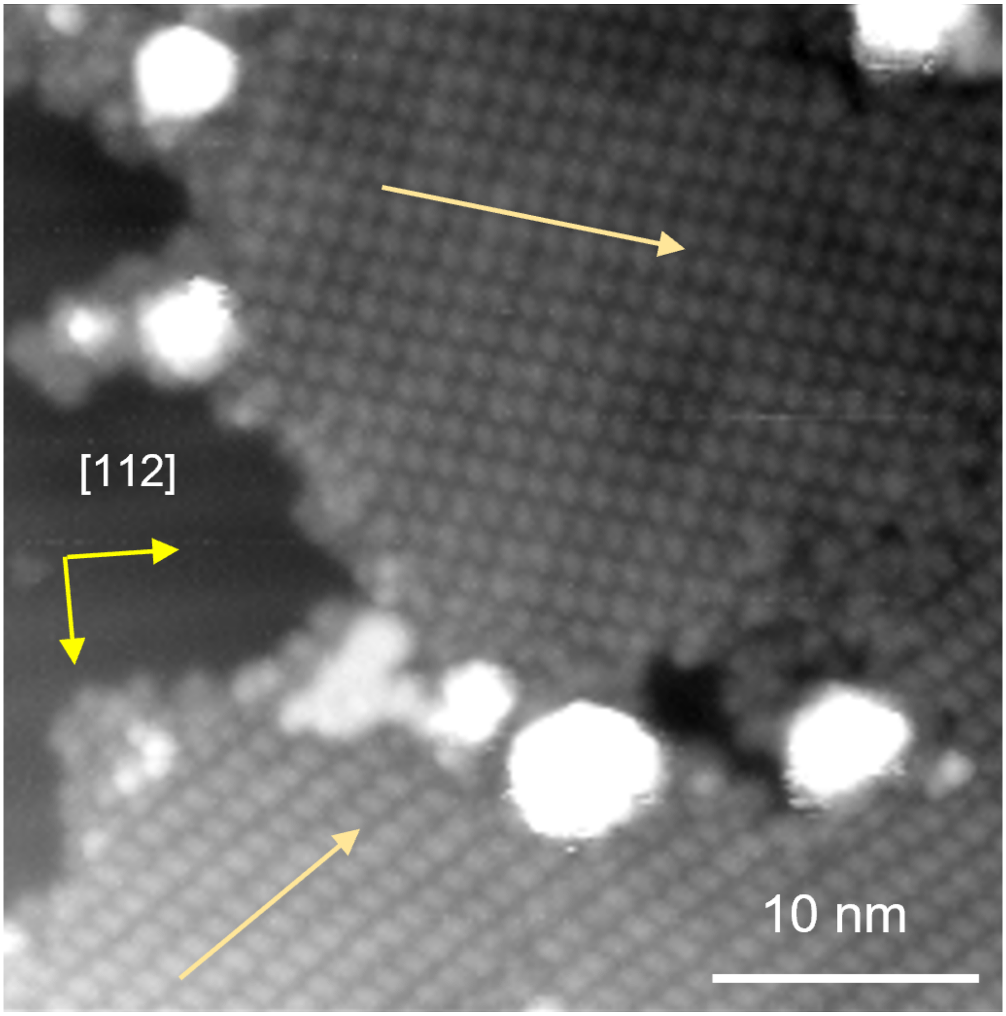

Figure S1: **The large-scale STM of NBDG.** The a-axis vector of the unit cell, indicated by the yellow arrow, is oriented at $-15.5 \pm 1°$ and $+35.5 \pm 1°$ relative to the $[11\bar{2}]$ direction of the substrate, represented by the white arrow, for the upper and lower domains, respectively.

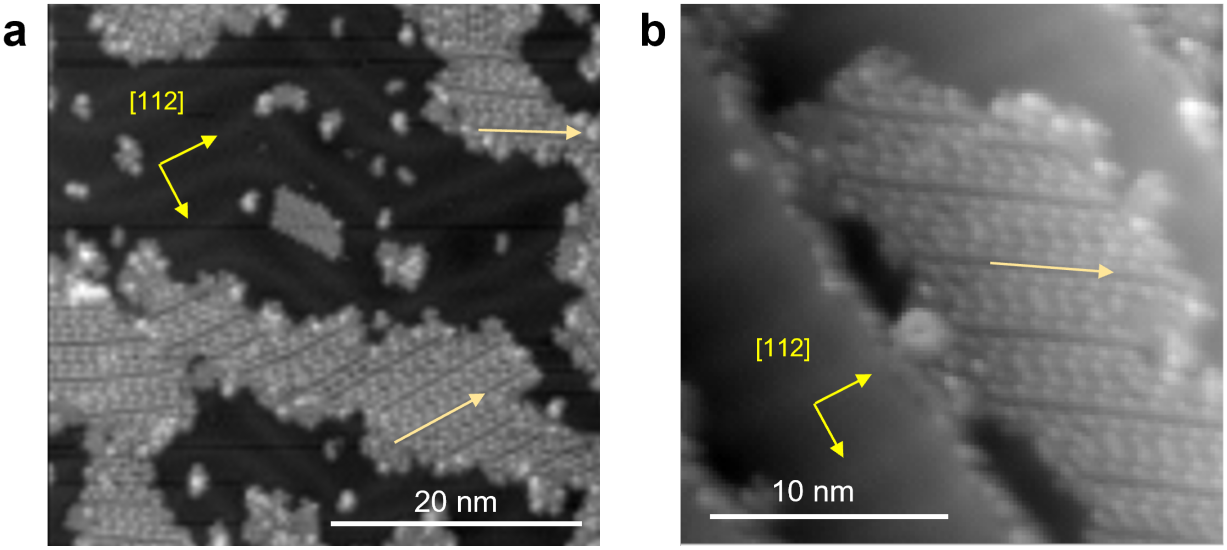

Figure S2: **The large-scale STM of NADG taken from different area. a**, The a-axis vector of the unit cell, indicated by the yellow arrow, is oriented at $+30.0 \pm 1°$ and $0.0 \pm 1°$ relative to the $[11\bar{2}]$ direction of the substrate, represented by the white arrow, for the upper and lower domains, respectively. **b**, The a-axis vector of the unit cell is oriented at $+30.0 \pm 1°$ relative to the $[11\bar{2}]$ direction of the substrate.

Supplementary experimental data: STM topography line profiles

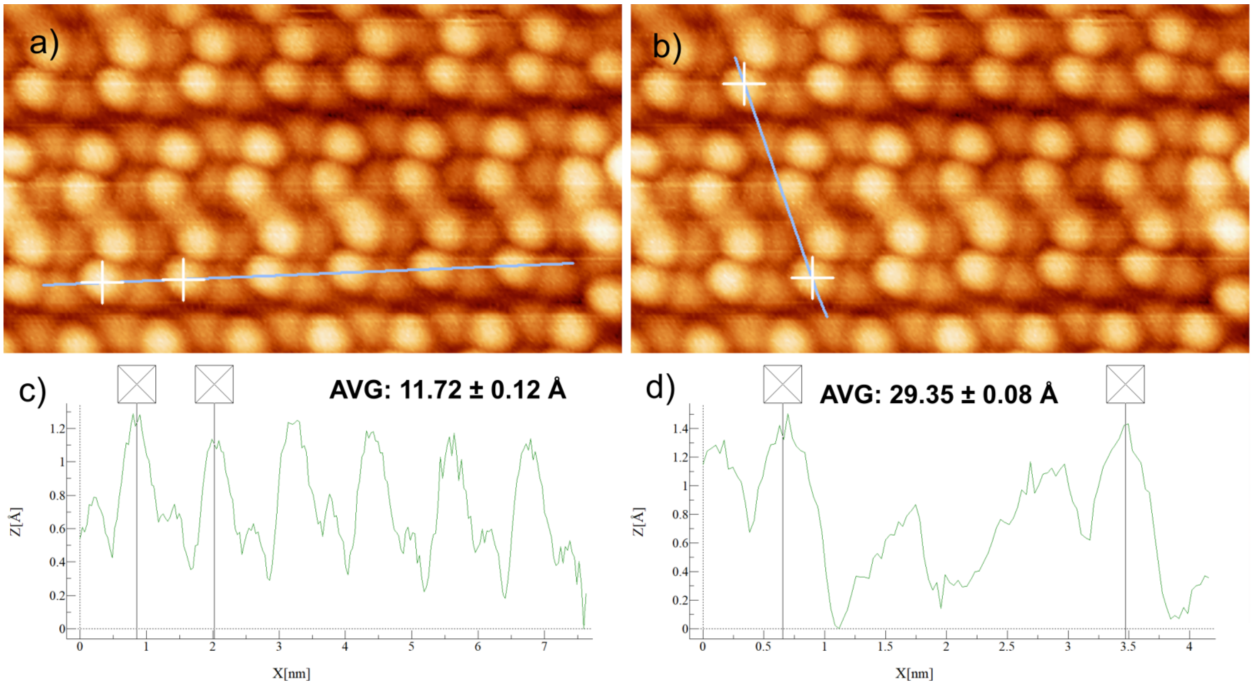

Figure S3: Determination of lattice parameters for the α-monolayer from STM line profiles. a) and c) shows the line profile for the a-vector, b) and d) shows the line profile for the b-vector. The angle between the two (γ) was found to be 107°.

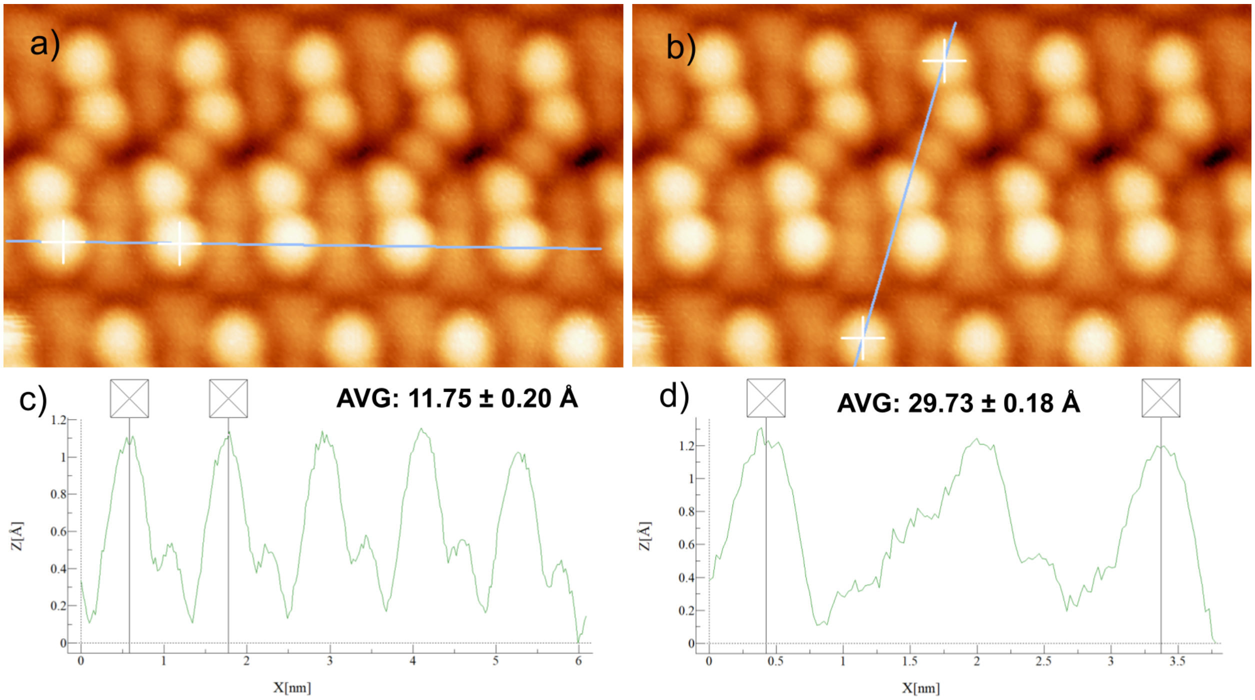

Figure S4: Determination of lattice parameters for the β-monolayer from STM line profiles. a) and c) shows the line profile for the a-vector, b) and d) shows the line profile for the b-vector. The angle between the two (γ) was found to be 75°.

The dimensions of the repeating units for NADG and NBDG self-assemblies were determined by averaging five repeating units in both the a- and b- directions as illustrated in Figures S3 and S4, the average dimensions for NADG being (a, b, γ) = (11.72 Å, 29.35 Å, 107°), the NBDG counterparts (a, b) = (11.75 Å, 29.73 Å, 75°), in fair agreement with the DFT-relaxed unit cell dimensions. However, the experimental b-vector is consistently smaller than the theoretical counterpart by 0.61 and 0.28 Å for NADG and NBDG.

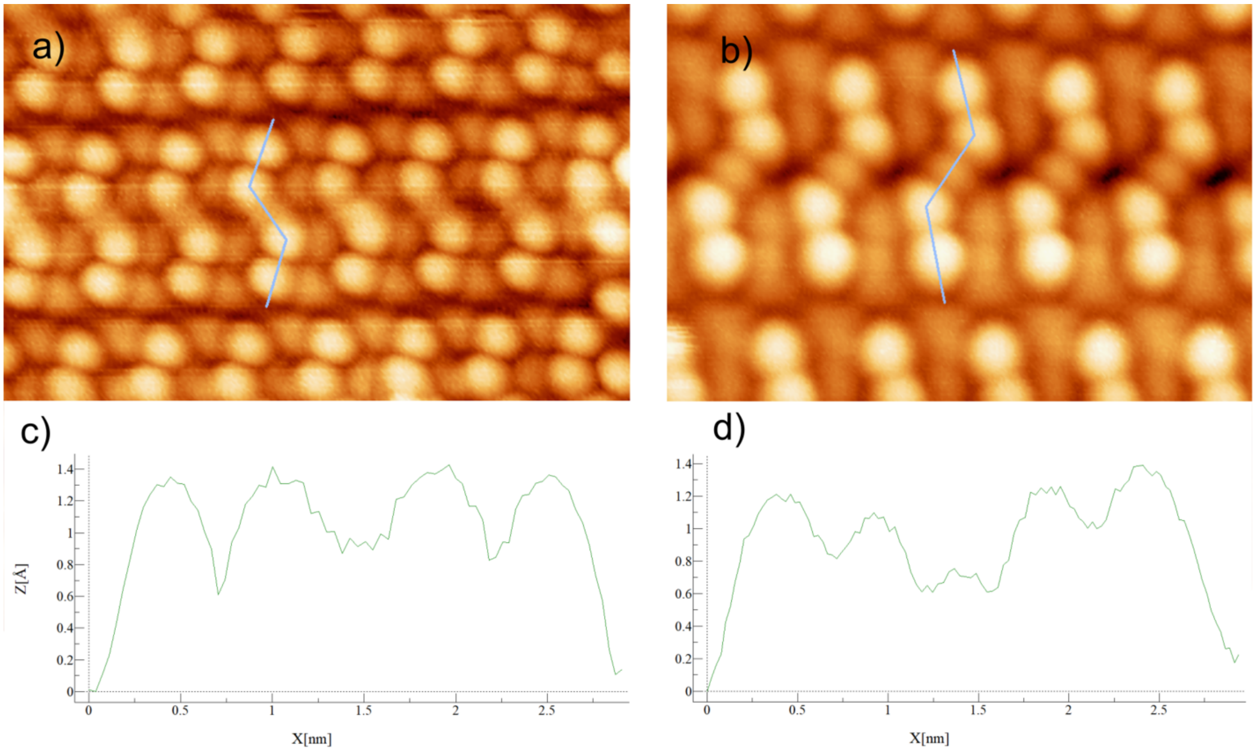

Figure S5: Line profiles along the highest parts of the repeating units for α- (a, c) and β-monolayers (b, d). The profiles are drawn from top to bottom based on the a) and b) images.

To gain further insight into the detailed structures of the monolayers, we looked at the line profiles across the parts that were protruding the furthest from the surface as illustrated in Figure S5. Both NADG and NBDG display similar profiles, with four higher protrusions in addition to a sligthly lower one corresponding to the area in between the two central units. However, we note one minor difference between the two assemblies, where the central units in NADG are slightly higher, or at similar heights than the edges, while the opposite is true for NBDG. These observations are consistent with the AFM images, with the center of NADG showing up as brighter features earlier in the AFM image height stack, while the NBDG structure display more sharp features at the edges than in the center.

Computational

Bayesian Optimization Structure Search (BOSS)

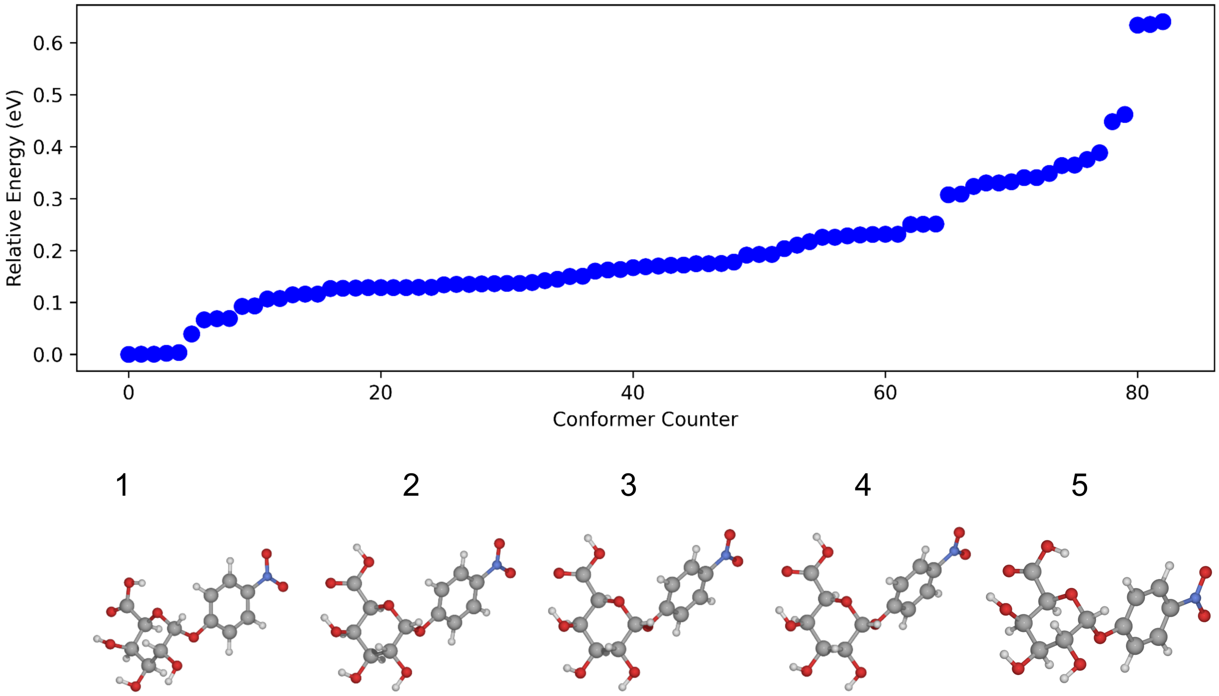

Figure S6: PBE+vdW^{surf} relaxed conformer energies and the five lowest energy α-anomer structures predicted by BOSS.

Figure S6 displays the results of the conformational analysis for the α-anomer of 4-nitrophenyl-D-galacturonide. The lowest energy structure has a clockwise H-bond network involving the -OH and -COOH groups the of the galacturonide moiety. While the hydrogen in the -COOH group is here in a *trans*-orientation towards the O_5-atom, an isoenergetic conformer has it in the *cis*-orientation, i.e. towards the carbonyl oxygen. A final intramolecular H-bond is formed between the O_5-atom and a ortho-hydrogen in the nitrophenyl group, located above the central galacturonide plane. The rest of the structures differ in the rotation of the nitrophenyl ring with respect to the galacturonide moiety.

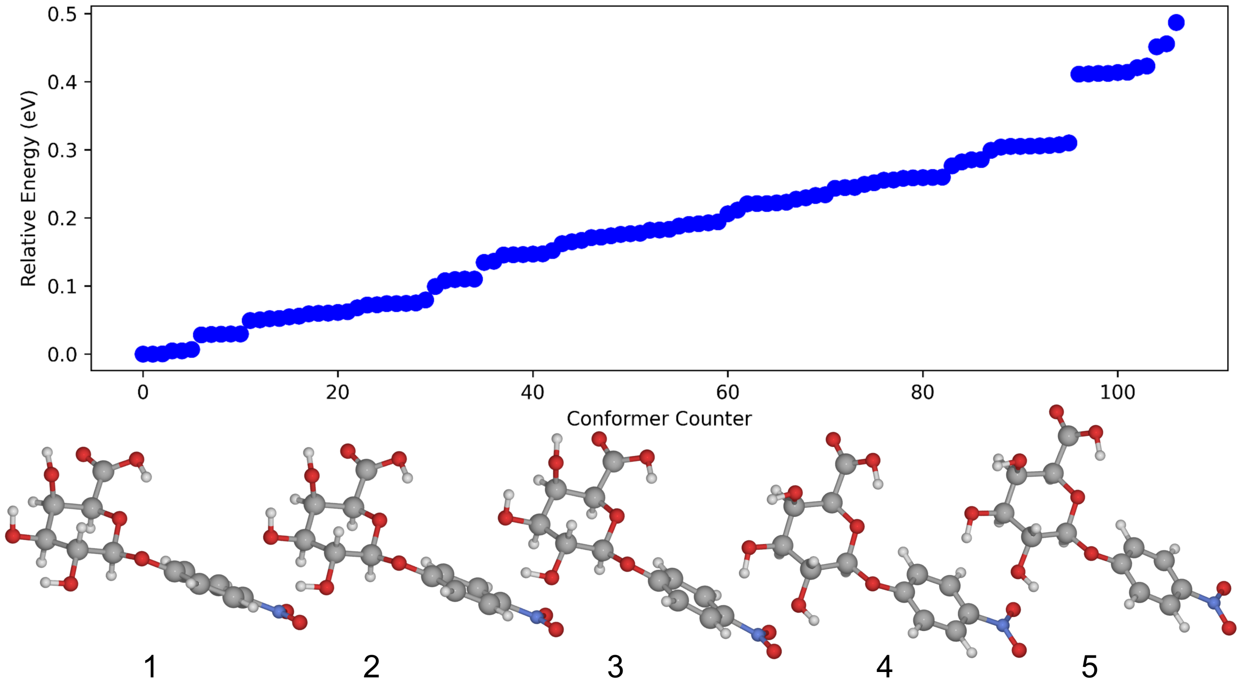

1 2 3 4 5

Figure S7: PBE+vdW^{surf} relaxed conformer energies and the five lowest energy β-anomer structures predicted by BOSS.

Figure S7 displays the results of the conformational analysis for the β-anomer of 4-nitrophenyl-D-galacturonide. The structures determined are similar to those for the corresponding α-anomer, with a similar clockwise H-bond network. However, due to the equatorial placement of the nitrophenyl group, the orientation of the latter makes the H-bond to the O_5-atom now appear below the central galacturonide plane. This is likely due to the above-plane alternative bringing the hydrogen on the nitrophenyl group too close to that of the COOH-group, with added repulsion compared to the below-plane bond. Furthermore, the fourth lowest β-anomer displays a counter-clockwise H-bond network 0.005 eV (0.5 kJ/mol) higher than the global minimum, while the α-counterpart has this as the sixth in line, 0.04 eV (4 kJ/mol) higher than the global minimum.

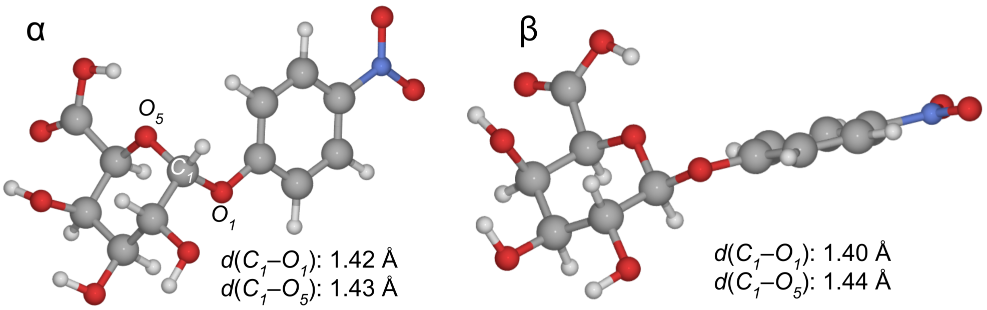

Figure S8: Global minimum conformers for isolated α- and β-anomers identified using BOSS. Relevant bond distances for the anomeric effect are shown below each molecule.

By analyzing the donor-acceptor interactions *via* second order perturbation theory in the natural bond orbital (NBO) basis[S1] for the global minimum conformers, we note that the endo and exo anomeric effects are balanced in the α-anomer, both providing around 50 kJ/mol stabilization, leading to nearly equal C_1–O_5 and C_1–O_1 bonds at 1.43 and 1.42 Å. Meanwhile, for the β-anomer, the exo anomeric effect is stronger than the endo anomeric effect, providing 58 and 16 kJ/mol of stabilization, respectively. This implies that for the latter, donation of the exocyclic oxygen lone pair ($n_{(O1)}$) to the σ^* orbital of the C_1–O_5 bond is more efficient than donation of the endocyclic oxygen lone pair ($n_{(O5)}$) to the C_1–O_1 σ^* orbital, which in turn is reflected in the corresponding bond distances at 1.44 and 1.40 Å, respectively. In addition, the difference in energy for the two anomers is computed to be 7 kJ/mol (PBE+vdW^{surf}), with the α-anomer being lowest, which is consistent with the anomeric effect stabilizing the α-anomer.

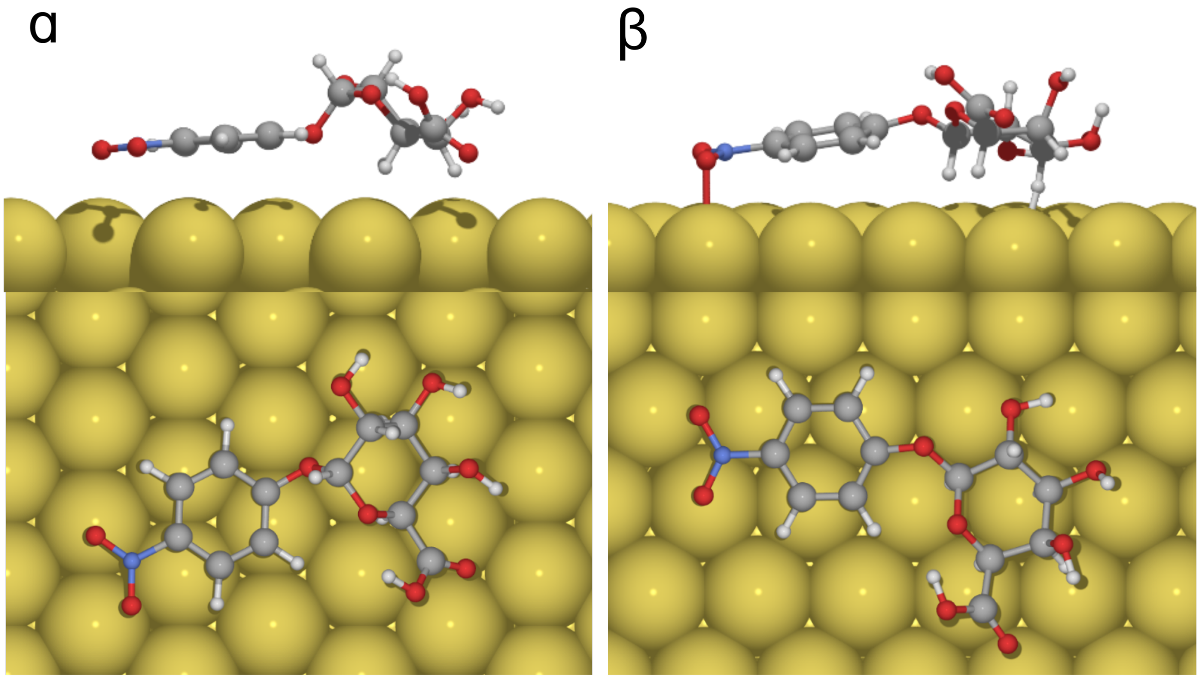

Figure S9: Global minimum PBE+vdW^{surf}-relaxed adsorption structures for single α- and β-anomer molecules as identified by BOSS.

Following the conformer analysis, the same approach was used to find the most stable adsorption configuration of single NADG and NBDG adsorbates, this time employing the global minimum isolated molecules as building blocks in the adsorption structure search, while the surface slab was acting as the second building block for BOSS. The surface slab for the single molecule adsorbates was constructed from four layers of 7×8 Au atoms in the fcc111 crystal structure. The most stable adsorption configurations of the single molecules determined by BOSS differ in that the glycosidic bond is pointing towards the surface in NADG, while it is pointed away from the surface in NBDG (Figure S9). The adsorption energies of the two are similar at −1.60 eV (−154.2 kJ/mol) for NADG, −1.65 eV (−159.5 kJ/mol) for NBDG, the difference being close to the magnitude of the anomeric effect stabilizing the NADG molecule. Meanwhile, flipping the NBDG molecule to orient its glycosidic bond towards the surface leads to an adsorption configuration only 0.10 eV (9.2 kJ/mol) higher in energy than the global minimum. Similarly, flipping over NADG costs 0.11 eV

(11.5 kJ/mol). Considering the multiple possible hydrogen bond donors and acceptors on each molecule, and the typical H–O$\cdots$H–O bond energy being around 0.22 eV (21 kJ/mol), the global minimum configurations of the single adsorbates are not necessary equivalent to those attained by the molecules in monolayer as a more favorable hydrogen bonding pattern might counterbalance the lower energy of a given rotational state of the molecule on the surface.

Minima hopping for monolayer structures

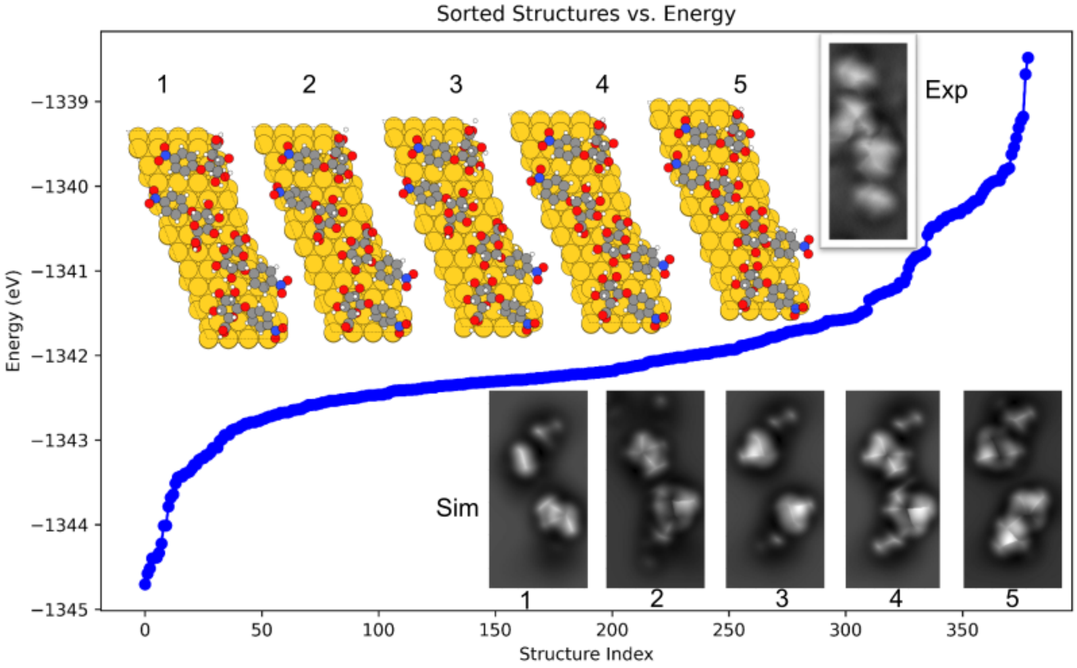

Figure S10: Global minimum monolayer structures for the α-anomer from minima hopping using NequIP and their simulated AFM images. Numbering indicates the relative energy of the structure, with increasing number corresponding to higher energy. Experimental AFM image shown as inset.

Initial monolayer structures were subsequently constructed by hand using the most stable single molecule adsorption configurations as building blocks, closely guided by the experimental constant current STM images. Subsequently, we simulated the AFM images of the initial 50 structures with the Probe-Particle Model,.[S2,S3] Here it became apparent that the structures were merely close enough to the experiment to recognize single features in the images. Furthermore, manual construction of the monolayer structures and the following DFT relaxation was a highly time-consuming and computationally expensive endeavor. To accelerate the screening of the required configurations, we trained an MLIP, NequIP,[S4] on the already acquired data, supplemented by high-temperature molecular dynamics snapshots from the semiempirical tight-binding DFT method GFN-xTB,[S5] subsequently recomputed

using the same DFT functional (PBE+vdW^{surf}) as the rest of the data entries. The purpose of the latter was to improve the reliability of the MLIP by including a more complete configurational phase space in the training data. With the trained MLIP, we used the minima hopping algorithm as outlined by Goedecker,[S6] placing Hookean constraints the adsorbates to preserve molecular identity and to restrict them from sampling irrelevant parts of the phase space, such as the vacuum region.[S7] Minima hopping was chosen here as BOSS is restricted to less than 20 degrees of freedom, which is sufficient for the conformer analysis and the isolated adsorbates, yet insufficient to establish the structures of the full monolayers. This sampling provided 850 monolayer structures in total for the two anomers, and along with them rough versions of the experimentally observed monolayer structures.

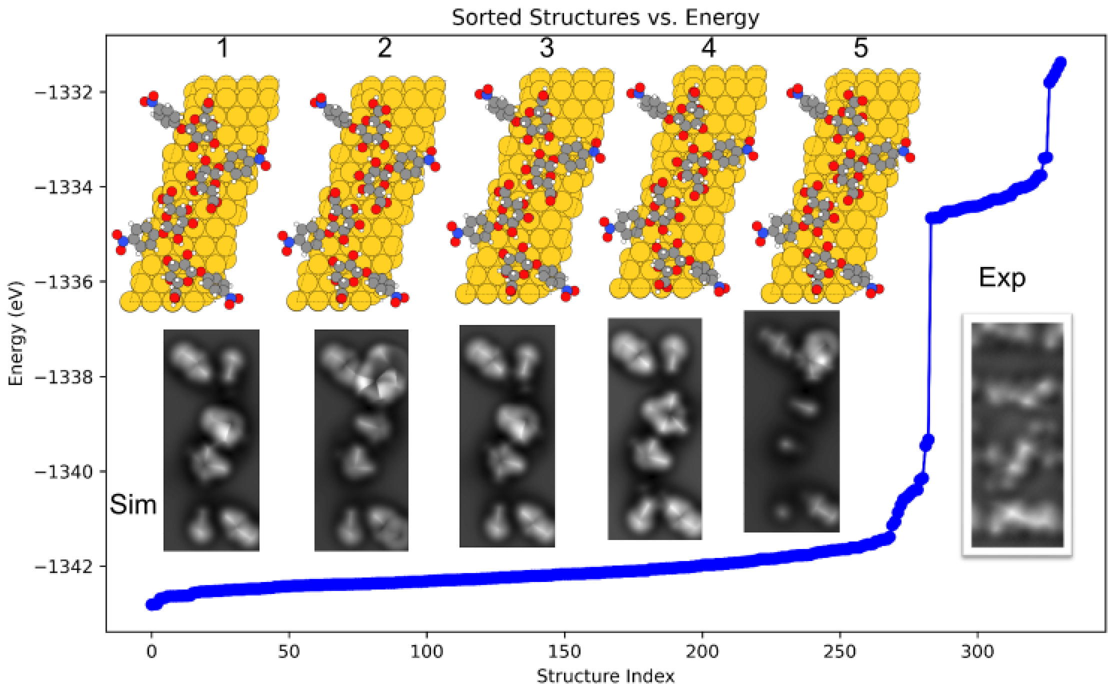

Figure S11: Global minimum monolayer structures for the β-anomer from minima hopping using NequIP and their simulated AFM images. Numbering indicates the relative energy of the structure, with increasing number corresponding to higher energy. Experimental AFM image shown as inset.

The global minimum structures provided by the minima hopping procedure are overall close to those imaged experimentally–in particular for NBDG–but with minor discrepancies

in functional group orientations and in the heights of specific atoms, which leads to noticeable differences in the simulated images. The final structures leading to the closest apparent match with experimental images were determined by altering the positions of mismatching atoms by hand, while fixing the atoms responsible for contrast similar to experiment. These altered structures were pre-relaxed with the MLIP before final DFT relaxations to reduce the amount of time spent on the latter. It should be noted that the final structures were kept partially constrained during relaxation. Specifically, the atoms surrounding the carboxyl groups of the central monosaccharide units of both structures were fixed in a position higher than in the relaxed counterparts. This suggests that there could be some feature in the surface which we were unable to capture with our methods. This could for instance be adatoms, an assumption supported by the fact that the monolayers are formed close to step edges, potentially acting as a source of adatoms. It has also been shown that organic molecules deposited onto a surface with solvent, such as methanol, may expedite adatom migration relative to UHV deposition, consistent with the above hypothesis.[S8] A second possibility is that the electrospray deposition process itself introduces either solvent molecules or contaminants which could be incorporated into the monolayer structure, such as Na or Ca ions, known to leach from laboratory glassware.[S9] A final third option could be that the slightly elongated units cells of our model structures do not fully capture the packing effects due to neighboring adsorbates, with the real systems being slightly more crowded than our models.

Although we initially assumed that the interactions between pairs of carboxyl groups would be a key driving force in the self assembly process, our structural models contradicts this. Specifically, a highly symmetric configuration with two O–H$\cdots$O=C contacts –akin to the most stable formic acid dimer– is completely absent here, which we assume to be closely related to the most efficient packing of the adsorbates on the surface. However, we do observe a pair of closely related asymmetric structural motifs in the center of NADG, with one O–H$\cdots$O–H and one C=O$\cdots$H–C contact. These results can be rationalized using

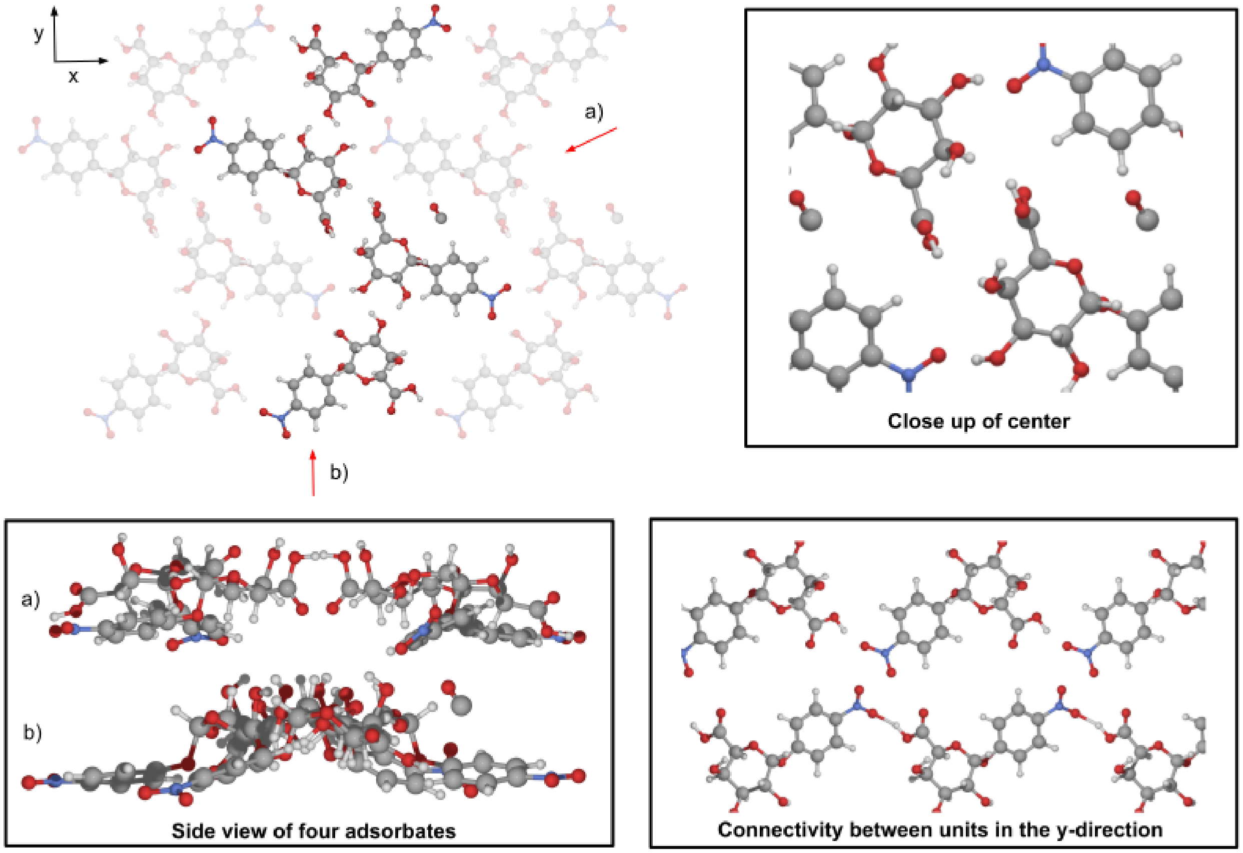

Figure S12: Final structural model of the NADG assembly.

the formic acid dimer as a prototypical model of the interactions of the carboxyl groups in our systems, noting how the latter is analogous to the fourth most stable formic acid dimer structure.[S10,S11] Based on accurate quantum chemical computations,[S10] the interaction energy of the symmetric formic acid dimer is 53.7 kJ/mol, while that of the asymmetric motif is 23.3 kJ/mol. Hence, forming two of the latter does not quite balance out the stronger interaction of the symmetric structure in this model system. However, as shown in the structural model of the NADG monolayer in Figure S12, this asymmetric bonding motif involves the hydroxymethyl -OH groups in a cyclic hydrogen bond network with the -OH groups of the α-carboxyl, which is likely strengthened by co-operativity effects.[S12–S14] This cyclic network is directly observable in the AFM images, responsible for the contrast of the central features in the far and mid tip-distance images shown in Figure S13. In addition to these local effects, the asymmetric structure allows a more close packing of the central adsorbates, which extends to the full monolayer.

In the final NADG structural model, a CO molecule was included between the 1D central units in the propagation direction to account for this seemingly isolated feature in the experimental AFM image. We note that this is not present between each repeating unit.

Large-scale AFM overlap

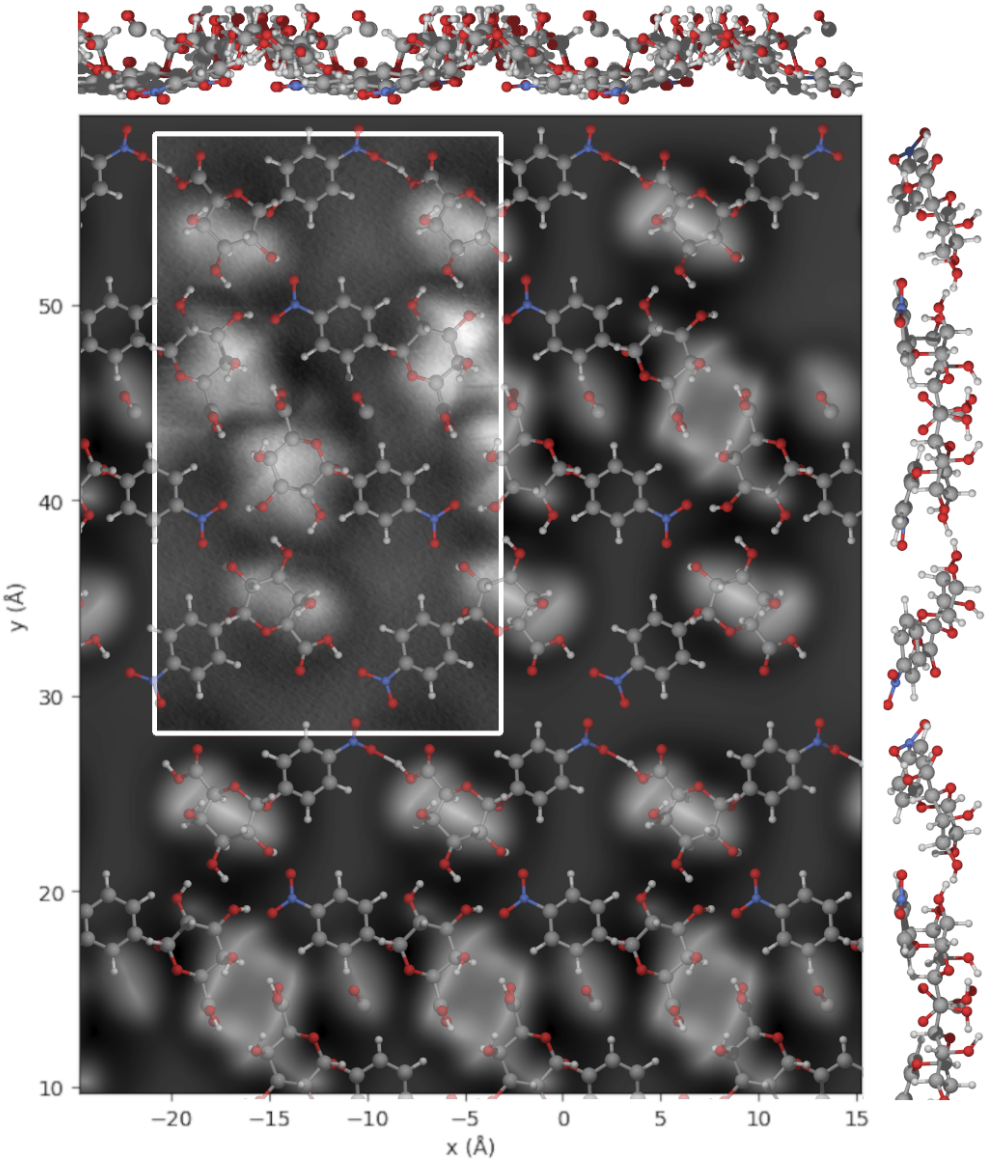

Figure S13: Comparison of simulated (larger image) and experimental (inset) AFM images with superimposed molecular structures for the α-anomer.

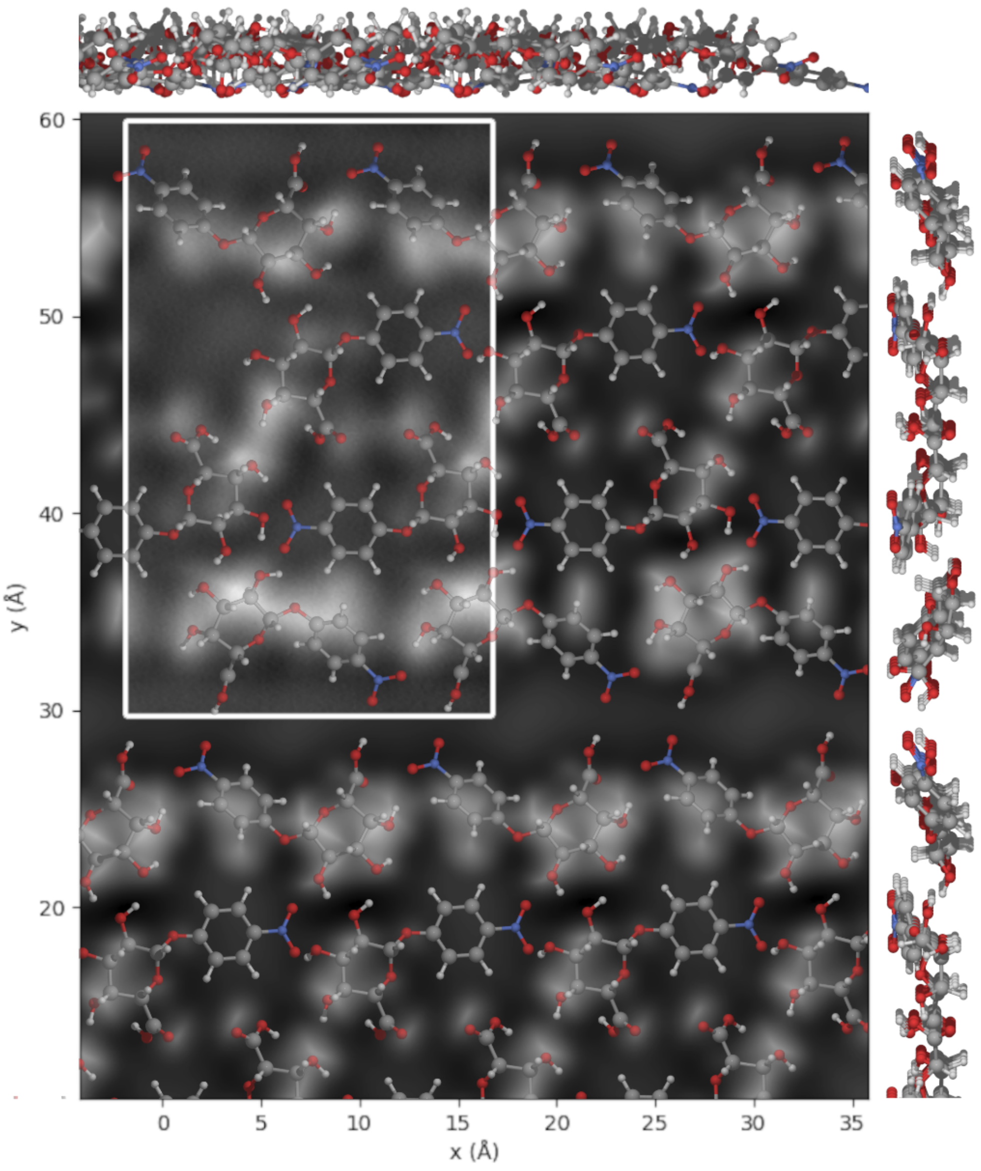

Figure S14: Comparison of simulated (larger image) and experimental (inset) AFM images with superimposed molecular structures for the β-anomer.

Comparison of experimental and simulated STM and AFM images

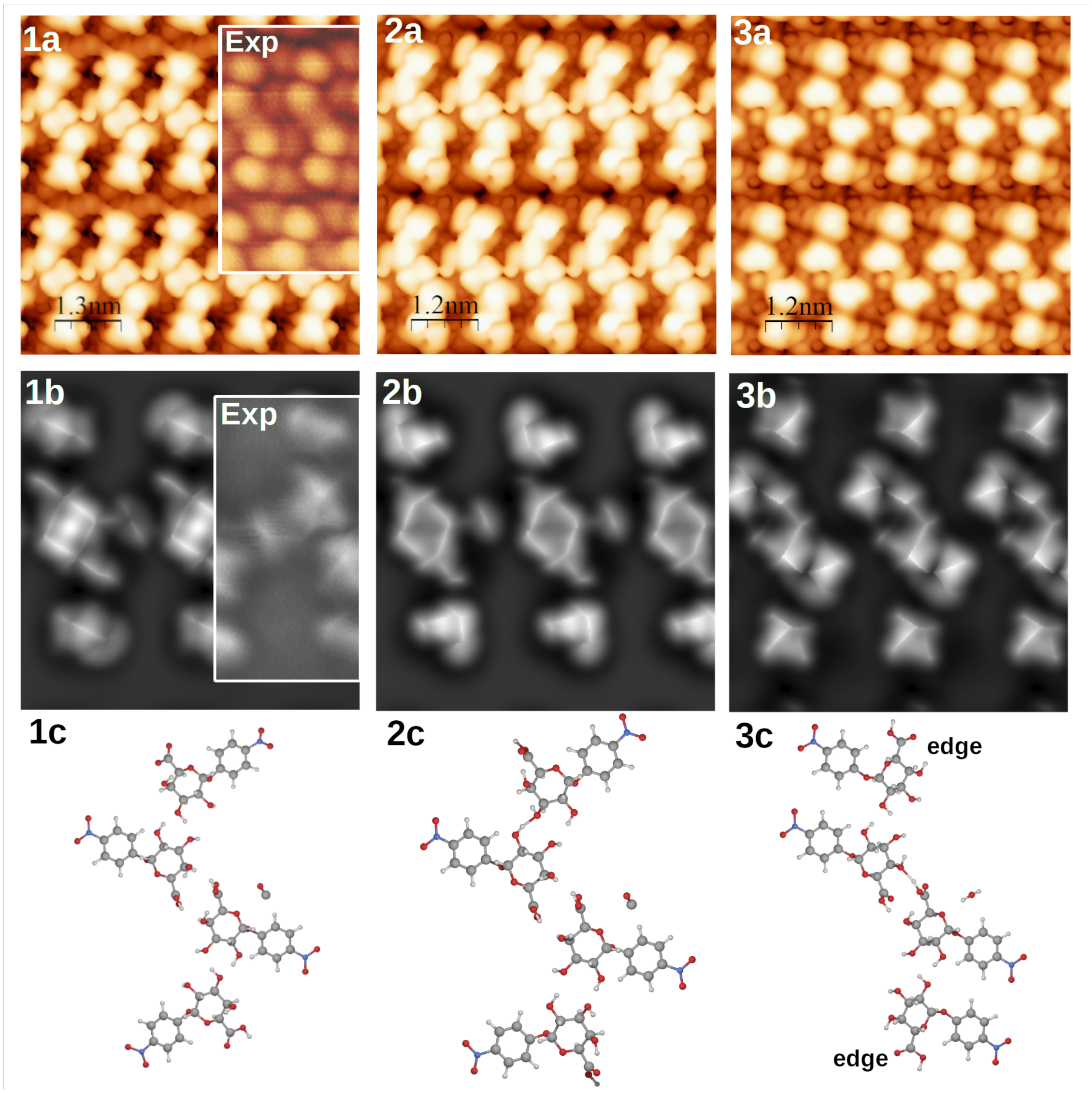

Figure S15: A selection of α-monolayer structures (1c-3c) and their simulated STM (a) and AFM (b) images. Experimental STM and AFM images are shown in 1a and 1b as insets.

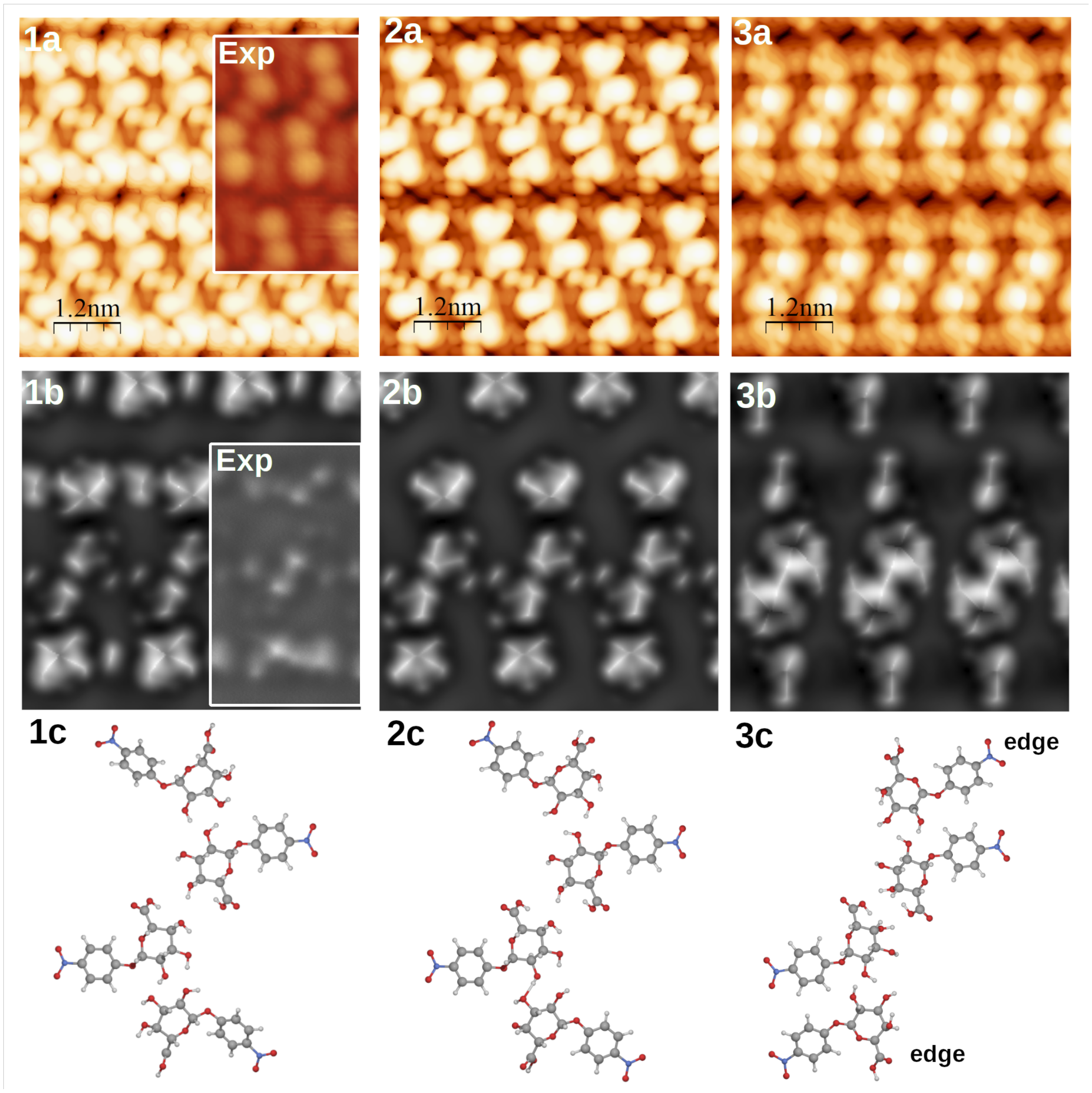

Figure S16: A selection of β-monolayer structures (1c-3c) and their simulated STM (a) and AFM (b) images. Experimental STM and AFM images are shown in 1a and 1b as insets.

Rotational barriers of hydroxyl and carboxylate groups in the NADG assembly

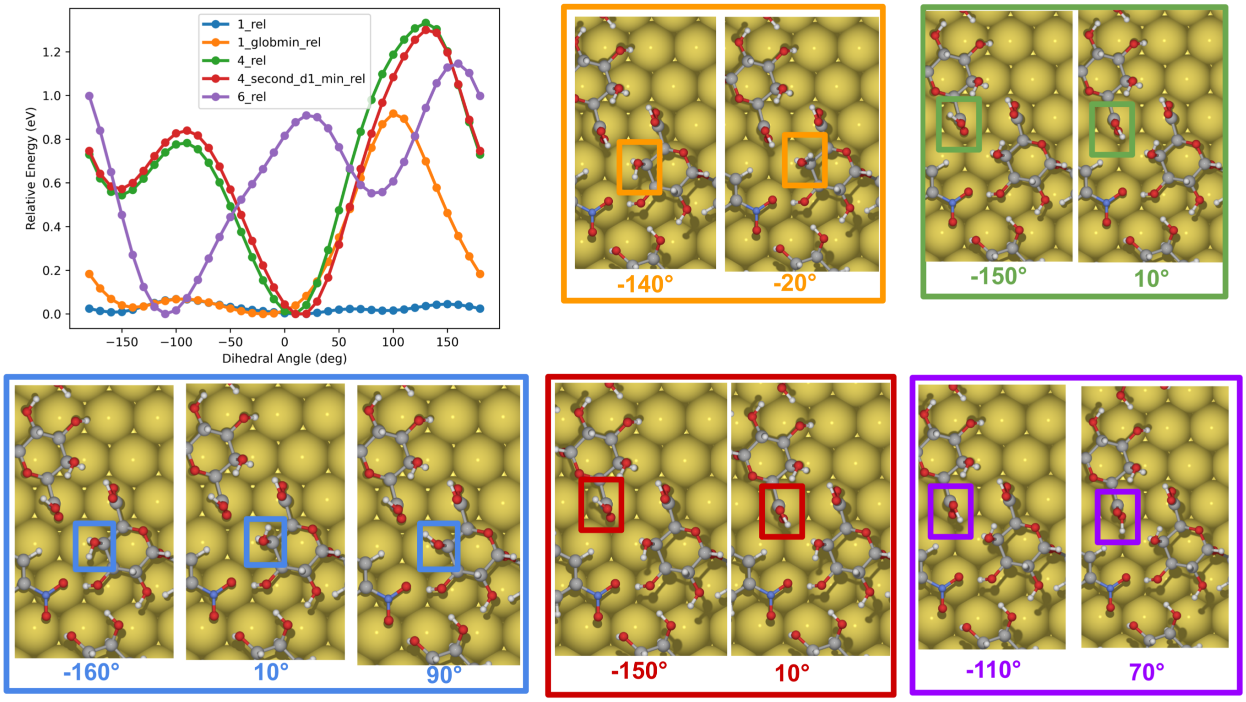

Figure S17: Energy curves for -OH rotation in the center of the NADG assembly. Orange marks the rotation of the bottom/top -OH groups (d1) with the dihedral angles of the right/left -OH groups kept identical to the experimentally determined model structure. Blue marks the rotation of bottom/top -OH groups (d4) with d1 $= -150°$. Green marks the rotation of the right/left -OH groups with the bottom/top groups kept identical to the experimentally determined model structure. Red marks the rotation of the right/left -OH groups with d1 $= -140°$. Purple marks the rotation of the right/left -COOH groups (d6) with all -OH groups kept identical to the experimentally determined model structure.

To further investigate the effects of flexible groups of the sample on the image contrast of the NADG structure, we scanned the DFT energy for the rotation of the -OH groups in the central molecules of the monolayer as shown in Figure S17. This was done due to the characteristic features appearing here at close tip-sample distances. Starting from the model structure corresponding the simulated images in the main manuscript, we scanned the DFT energies corresponding to rotation of the left (d4, in green) and bottom (d1, in orange) -OH groups, which are equivalent to the right and top groups by symmetry. Here we find that

perturbing the bottom -OH group has two minima at d1 $= -150°$ and $-20°$, separated by a 0.1 eV barrier. This indicates that the -OH groups at the top and bottom of the central structure are quite flexible, having a range of 130 degrees of rotation within a small energy window. We also scanned the rotations of the -OH groups while keeping the other -OH group in the higher-lying local minima, but found that the initial rotation of d1 is the most facile alteration of the structure as determined by the barriers, even though a smaller rotational barrier is found for the transition of d1 from 10° to 90°, displaying a computed barrier of 0.02 eV, this requires that d4 is first changed from 10° to $-150°$, the two latter minima being separated by a 0.8 eV barrier. However, this analysis assumes that single bonds are rotated in isolation, while in reality the whole system hydrogen-bonded network are affected by localized perturbations in the structure, meaning that this analysis is just a simple model trying to explain the contrast changes at close tip-sample distances. We also estimated the rotational forces by numerical derivation of the DFT potential energy U:

$$F = -dU/\theta$$

as well as the activation forces ($F_{activation}$) required to surmount the energy barriers for the transitions between local rotational minima using the linear distance (x_{linear}) the hydrogen has to traverse around the oxygen atom,

$$x_{linear} = (2\pi \cdot r \cdot \theta)/360$$

$$F_{activation} = \Delta E / x_{linear}$$

where the ΔE is the energy barrier height in J, r is the O–H bond distance in m, θ is the angle of rotation between the minimum and the barrier. These are found to vary between 0.05 – 1.2 nN for the lowest and highest OH-rotational barriers, respectively. To facilitate comparison with the bending stiffness of the CO tip (typical values of k = 0.24 –

0.5 N/m),[S15] we approximated the -OH and -COOH group rotational minima as harmonic oscillators to estimate their bending stiffness values as shown in Figure S18. This was done by fitting a second degree polynomial to the potential energy minima,

$$U = 1/2 \cdot kx_{linear}^2$$

Here we find force constants for the rotationally bound minima ranging from k = 4.4 – 19 N/m, which are about one and two orders of magnitude larger than the bending stiffness of the CO-tip. This suggests that the bending of the CO tip alone is not enough to perturb these functional groups significantly, but given close enough tip-sample distances, the C–O stretching component could play a significant role with a force constant around 1860 N/m for the isolated molecule. Furthermore, the rotation stiffness of the -COOH group is slightly smaller than the rotation of the left/right -OH groups, which are bound to it, which implies that tip-induced rotation of the latter is possibly accompanied by rotation of the former to some extent.

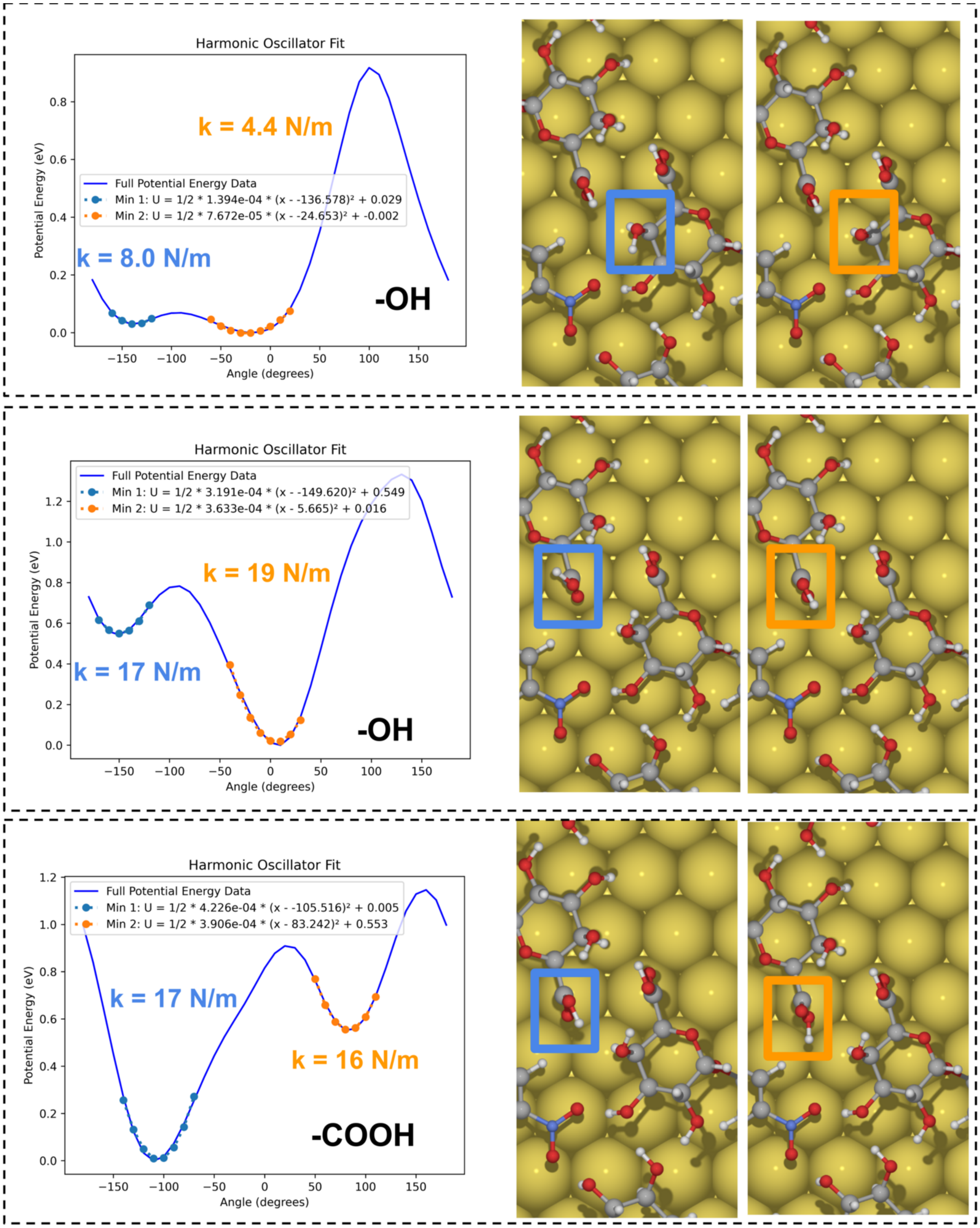

Figure S18: Determination of force constants from the -OH and -COOH group rotational motion bending potentials. The structures shown correspond to the minima in the potential energy plots as indicated by coloring. Note the different energy scales on the figures.

Orbital interactions

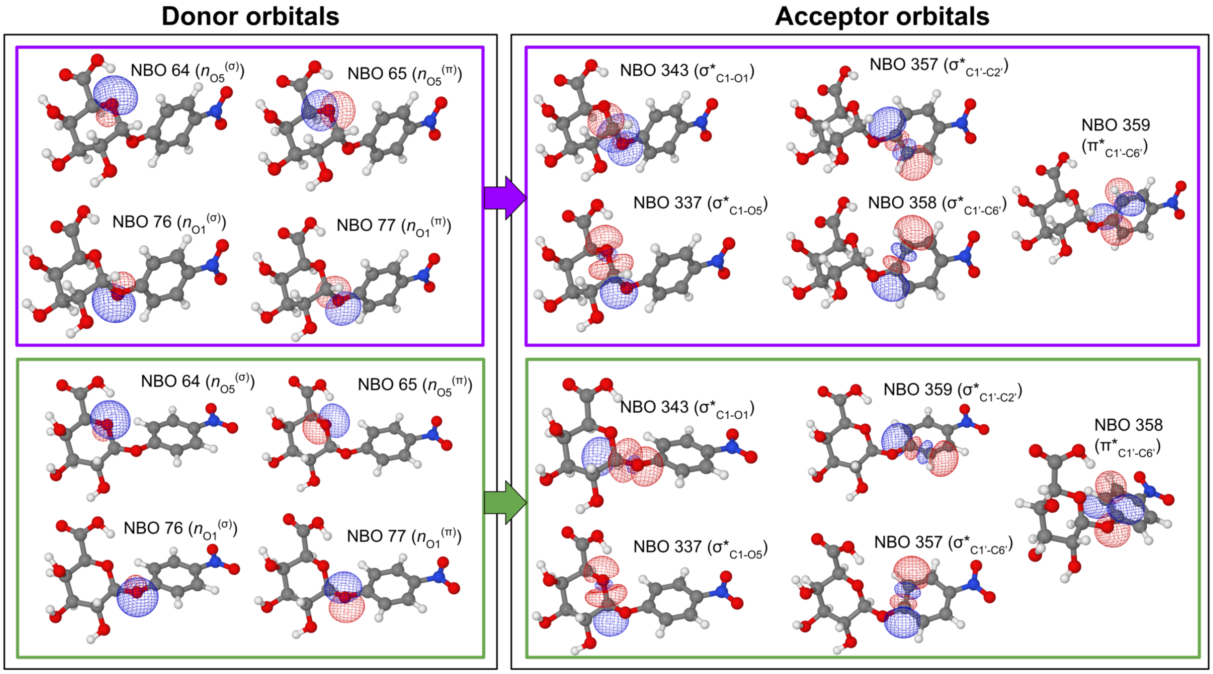

Figure S19: Overview of donor and acceptor orbitals from Natural Bond Orbital (NBO) analysis. The shown orbitals correspond to the pre-orthogonal Natural Bond Orbitals (PNBO), which retains the symmetry of the component atomic orbitals for intuitive visualization and analysis. Orbitals in purple boxes correspond to the α-anomer, green to the β-anomer.

The donor-acceptor orbital interactions relevant to the anomeric effects for **NADG** and **NBDG** are displayed in Figure S19, computed using the NBO method in Gaussian.[S16,S17] Typically, one considers the O_5/O_1 lone pair and the antibonding C_1–O_1/C_1–O_5 orbitals the most important for the anomeric effect. These correspond to NBOs 64, 65, 76, 77 for the O_5/O_1 σ- and π-lone pairs as donors, while the C_1–O_1/C_1–O_5 antibonding orbitals are described by NBOs 343 and 337 as acceptors, respectively. Furthermore, we find that interactions with σ^*- and π^*-orbitals in the nitrophenyl groups, NBOs 357, 358 and 359 are significant for the overall anomeric effect in the assembled structures, specifically by providing alternative acceptor sites for the O_1-lone pairs.

Charge density differences

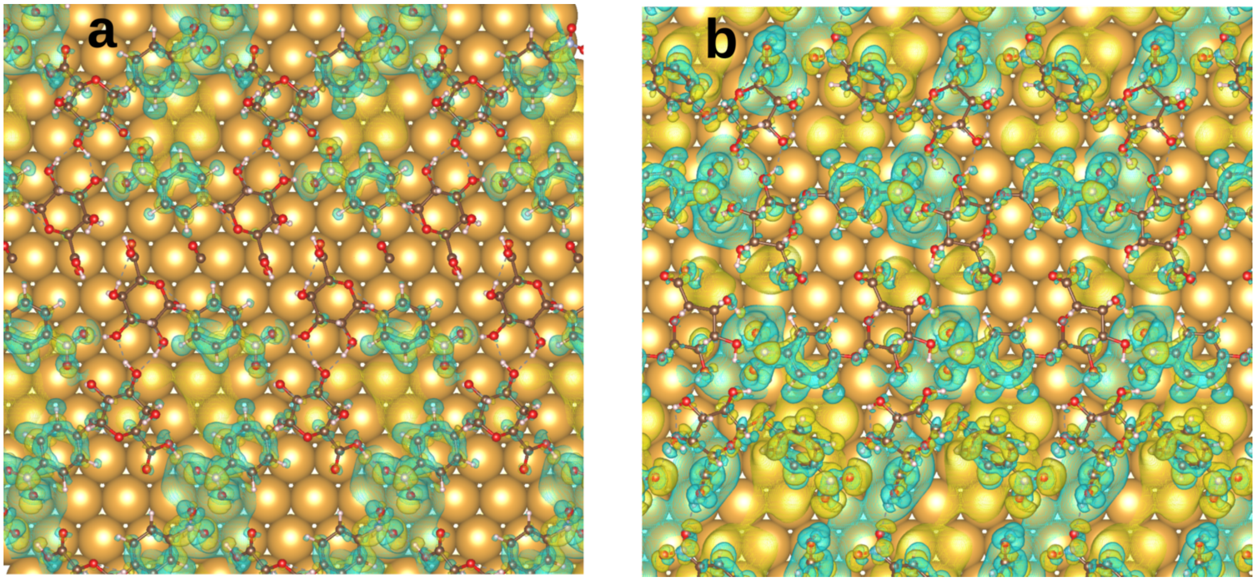

Figure S20: Charge density differences between the full system and the isolated substrate and molecules. for the α (a) and β (b) self-assemblies computed with PBE+vdW^{surf}. Yellow isosurfaces correspond to positive electron density change (increased electron density), while blue correspond to negative changes (decreased electron density), isovalue = 0.0013.

References

(S1) Weinhold, F.; Landis, C.; Glendening, E. What is NBO analysis and how is it useful? *Int. Rev. Phys. Chem.* **2016**, *35*, 399–440.

(S2) Hapala, P.; Kichin, G.; Wagner, C.; Tautz, F. S.; Temirov, R.; Jelínek, P. Mechanism of high-resolution STM/AFM imaging with functionalized tips. *Phys. Rev. B* **2014**, *90*, 085421.

(S3) Krejčí, O.; Hapala, P.; Ondráček, M.; Jelínek, P. Principles and simulations of high-resolution STM imaging with a flexible tip apex. *Phys. Rev. B* **2017**, *95*, 045407.

(S4) Batzner, S.; Musaelian, A.; Sun, L.; Geiger, M.; Mailoa, J. P.; Kornbluth, M.; Molinari, N.; Smidt, T. E.; Kozinsky, B. E(3)-equivariant graph neural networks for data-efficient and accurate interatomic potentials. *Nat. Commun.* **2022**, *13*, 2453.

(S5) Grimme, S.; Bannwarth, C.; Caldeweyher, E.; Pisarek, J.; Hansen, A. A general intermolecular force field based on tight-binding quantum chemical calculations. *J. Chem. Phys.* **2017**, *147*, 161708.

(S6) Goedecker, S. Minima hopping: An efficient search method for the global minimum of the potential energy surface of complex molecular systems. *J. Chem. Phys.* **2004**, *120*, 9911–9917.

(S7) Peterson, A. A. Global Optimization of Adsorbate–Surface Structures While Preserving Molecular Identity. *Top. Catal.* **2014**, *57*, 40–53.

(S8) Ghalgaoui, A.; Doudin, N.; Sterrer, M. Nanostructuring of Au(111) during the Adsorption of an Aromatic Isocyanide from Solution. *Langmuir* **2017**, *33*, 91–99.

(S9) Ledieu, A.; Devreux, F.; Barboux, P.; Sicard, L.; Spalla, O. Leaching of borosilicate glasses. I. Experiments. *J. Non-Cryst. Solids* **2004**, *343*, 3–12.

(S10) Farfán, P.; Echeverri, A.; Diaz, E.; Tapia, J. D.; Gómez, S.; Restrepo, A. Dimers of formic acid: Structures, stability, and double proton transfer. *J. Chem. Phys.* **2017**, *147*, 044312.

(S11) Rodziewicz, P.; Doltsinis, N. L. Formic Acid Dimerization: Evidence for Species Diversity from First Principles Simulations. *J. Phys. Chem. A* **2009**, *113*, 6266–6274.

(S12) Fonseca Guerra, C.; Zijlstra, H.; Paragi, G.; Bickelhaupt, F. M. Telomere Structure and Stability: Covalency in Hydrogen Bonds, Not Resonance Assistance, Causes Cooperativity in Guanine Quartets. *Chem. - Eur. J.* **2011**, *17*, 12612–12622.

(S13) Nochebuena, J.; Cuautli, C.; Ireta, J. Origin of cooperativity in hydrogen bonding. *Phys. Chem. Chem. Phys.* **2017**, *19*, 15256–15263.

(S14) Trevisan, L.; Bond, A. D.; Hunter, C. A. Quantitative Measurement of Cooperativity in H-Bonded Networks. *J. Am. Chem. Soc.* **2022**, *144*, 19499–19507.

(S15) Hapala, P.; Švec, M.; Stetsovych, O.; van der Heijden, N. J.; Ondráček, M.; van der Lit, J.; Mutombo, P.; Swart, I.; Jelínek, P. Mapping the electrostatic force field of single molecules from high-resolution scanning probe images. *Nat. Commun.* **2016**, *7*, 11560.

(S16) Glendening, E. D.; Reed, A. E.; Carpenter, J. E.; Weinhold, F. NBO Version 3.1.

(S17) Frisch, M. J.; Trucks, G. W.; Schlegel, H. B.; Scuseria, G. E.; Robb, M. A.; Cheeseman, J. R.; Scalmani, G.; Barone, V.; Petersson, G. A.; Nakatsuji, H.; Li, X.; Caricato, M.; Marenich, A. V.; Bloino, J.; Janesko, B. G.; Gomperts, R.; Mennucci, B.; Hratchian, H. P.; Ortiz, J. V.; Izmaylov, A. F.; Sonnenberg, J. L.; Williams-Young, D.; Ding, F.; Lipparini, F.; Egidi, F.; Goings, J.; Peng, B.; Petrone, A.; Henderson, T.; Ranasinghe, D.; Zakrzewski, V. G.; Gao, J.; Rega, N.; Zheng, G.; Liang, W.; Hada, M.; Ehara, M.; Toyota, K.; Fukuda, R.; Hasegawa, J.; Ishida, M.;

Nakajima, T.; Honda, Y.; Kitao, O.; Nakai, H.; Vreven, T.; Throssell, K.; Montgomery, J. A., Jr.; Peralta, J. E.; Ogliaro, F.; Bearpark, M. J.; Heyd, J. J.; Brothers, E. N.; Kudin, K. N.; Staroverov, V. N.; Keith, T. A.; Kobayashi, R.; Normand, J.; Raghavachari, K.; Rendell, A. P.; Burant, J. C.; Iyengar, S. S.; Tomasi, J.; Cossi, M.; Millam, J. M.; Klene, M.; Adamo, C.; Cammi, R.; Ochterski, J. W.; Martin, R. L.; Morokuma, K.; Farkas, O.; Foresman, J. B.; Fox, D. J. Gaussian 16 Revision C.02. 2016; Gaussian Inc. Wallingford CT.
